# Association between glucagon‐like peptide‐1 receptor agonists and risk of dementia in older adults with type 2 diabetes: A target trial emulation

**DOI:** 10.1111/dom.70384

**Published:** 2025-12-22

**Authors:** Ting Zhou, Huilin Tang, Bingyu Zhang, Dazheng Zhang, Yiwen Lu, Lu Li, Jiajie Chen, Yong Chen, David A. Asch, Yong Chen

**Affiliations:** ^1^ The Center for Health AI and Synthesis of Evidence (CHASE), University of Pennsylvania Philadelphia Pennsylvania USA; ^2^ Department of Biostatistics, Epidemiology, and Informatics University of Pennsylvania Perelman School of Medicine Philadelphia Pennsylvania USA; ^3^ The Graduate Group in Applied Mathematics and Computational Science, School of Arts and Sciences, University of Pennsylvania Philadelphia Pennsylvania USA; ^4^ Pfizer New York City New York USA; ^5^ Leonard Davis Institute of Health Economics, University of Pennsylvania Philadelphia Pennsylvania USA; ^6^ Division of General Internal Medicine, Department of Medicine Perelman School of Medicine, University of Pennsylvania Philadelphia Pennsylvania USA; ^7^ Penn Medicine Center for Evidence‐Based Practice (CEP), University of Pennsylvania Philadelphia Pennsylvania USA; ^8^ Penn Institute for Biomedical Informatics (IBI), University of Pennsylvania Philadelphia Pennsylvania USA

**Keywords:** antidiabetic drug, cohort study, elderly, GLP‐1 analogue, pharmaco‐epidemiology, real‐world evidence

## Abstract

**Aim:**

Type 2 diabetes (T2D) is associated with increased dementia risk, but comparative data across newer glucose‐lowering therapies remain limited. We examined whether the initiation of GLP‐1 receptor agonists (GLP‐1 RAs) was associated with incident dementia compared with DPP4 inhibitors (DPP4is) and SGLT2 inhibitors (SGLT2is) in older adults with T2D.

**Materials and methods:**

We conducted a retrospective cohort study emulating a target trial using electronic health records from the University of Pennsylvania Health System (2019–2024), with external validation in the TriNetX U.S. Collaborative Network. Adults aged ≥50 with T2D, no prior dementia, and no GLP‐1 RA, DPP4i, or SGLT2i use in the previous year were eligible. New GLP‐1 RA users were propensity‐score matched (1:1) to DPP4i users (*n* = 6677 pairs) and SGLT2i users (*n* = 8434 pairs). The outcome was incident dementia, defined by ICD‐10‐CM codes. Cox proportional hazards models estimated hazard ratios (HRs), with multiple sensitivity and subgroup analyses performed.

**Results:**

Over a median follow‐up of 3.0 years (GLP‐1 RA vs. DPP4i) and 2.4 years (GLP‐1 RA vs. SGLT2i), GLP‐1 RA use was associated with a lower dementia risk versus DPP4is (109 vs. 148 events; HR 0.76, 95% CI 0.59–0.97), but a higher risk versus SGLT2is (127 vs. 64 events; HR 1.53, 95% CI 1.13–2.07). Findings were consistent across sensitivity and subgroup analyses. External validation confirmed reduced dementia risk versus DPP4is but not versus SGLT2is.

**Conclusions:**

In this large real‐world cohort, GLP‐1 RAs were associated with lower dementia risk than DPP4is but higher risk than SGLT2is. Prospective, biomarker‐informed studies are needed to clarify mechanisms and inform treatment choices in older adults with T2D.

## 1 INTRODUCTION

Type 2 diabetes (T2D) is associated with an increased risk of cognitive decline and dementia, likely through mechanisms involving vascular dysfunction, insulin resistance, and chronic inflammation, posing a significant public health challenge as the population ages.^1^, ^2^, ^3^ Epidemiologic studies indicate that individuals with T2D have a 1.5‐ to 2‐fold increased risk of developing dementia compared with those without diabetes.^4^, ^5^, ^6^ Concurrently, dementia has become a leading cause of disability and dependency among older adults worldwide, affecting an estimated 57 million people in 2021, a figure projected to increase by nearly 10 million annually.^7^ The global economic burden of dementia reached approximately US$1.3 trillion in 2019 and is projected to exceed $2 trillion by 2050, posing a significant challenge to healthcare systems worldwide.^8^ As the prevalence of both T2D and dementia continues to rise, identifying glucose‐lowering therapies that may also mitigate dementia risk has become a critical clinical and public health priority.

Glucagon‐like peptide‐1 receptor agonists (GLP‐1 RAs), initially approved for T2D treatment in 2005 and obesity management in 2014, have emerged as promising candidates for repurposing as disease‐modifying therapies in dementia.^9^, ^10^, ^11^ In addition to their glucose‐lowering properties, GLP‐1 RAs exhibit multiple neuroprotective effects, including attenuation of neuroinflammation, enhancement of synaptic plasticity, reduction in amyloid‐beta accumulation, and modulation of tau phosphorylation, key pathological features of dementia and Alzheimer's disease.^12^, ^13^, ^14^ Early‐phase clinical trials (e.g., ELAD, EVOKE, EVOKE Plus) and real‐world studies have suggested potential cognitive benefits of GLP‐1 RAs.^15^, ^16^, ^17^, ^18^ Furthermore, post hoc analyses from large cardiovascular outcome trials such as REWIND, LEADER, SUSTAIN 6, and PIONEER 6 have shown signals of cognitive benefit among GLP‐1 RA users.^16^, ^17^, ^19^ Despite these encouraging findings, real‐world comparative evidence on the cognitive effects of GLP‐1 RAs versus other frequently prescribed antidiabetic agents remains limited and inconclusive.

Sodium‐glucose cotransporter‐2 inhibitors (SGLT2is) and dipeptidyl peptidase‐4 inhibitors (DPP4is), two other widely used second‐line glucose‐lowering agents, have also been explored for their potential neuroprotective effects.^20^, ^21^ SGLT2is may confer cognitive benefits through mechanisms such as reduced neuroinflammation and oxidative stress,^20^ while DPP4is may enhance neuroendocrine signaling by inhibiting enzymatic degradation of incretin hormones.^21^ However, results from large‐scale clinical trials, including CARMELINA, have shown no significant reduction in cognitive outcomes with DPP4i use.^22^, ^23^ Although biologically plausible, the comparative effects of GLP‐1 RAs versus SGLT2is or DPP4is on dementia risk have not been definitively established in clinical practice, and findings from prior observational studies remain inconsistent.^22^, ^23^, ^24^, ^25^, ^26^, ^27^, ^28^, ^29^, ^30^, ^31^, ^32^, ^33^

To address these gaps, we conducted a retrospective cohort study emulating a target trial to assess the association between initiation of GLP‐1 RAs and incident dementia among older adults with T2D. Using electronic health record (EHR) data from the Penn Medicine health system, we compared GLP‐1 RA use to DPP4is and SGLT2is, applying rigorous methods to emulate the design and analysis of a hypothetical RCT.^34^ We applied propensity score (PS) matching and inverse probability treatment weighting (IPTW) to control for confounding, conducted competing risk analyses, externally validated findings using the TriNetX platform,^35^ and performed meta‐analysis to enhance generalizability. This study aimed to generate real‐world comparative evidence on the cognitive effects of GLP‐1 RAs and to identify patient subgroups most likely to benefit from their potential neuroprotective properties. By directly comparing the dementia risk associated with GLP‐1 RAs versus DPP4is and SGLT2is in routine clinical practice, our findings seek to inform more personalized diabetes management strategies for older adults at risk of cognitive decline.

## 2 METHODS

### 2.1 Study design and population

We conducted a target trial emulation study using EHRs from the University of Pennsylvania Health System (Penn Medicine), spanning 1 January 2019 to 30 September 2024. This data repository contains extensive real‐world data from the Hospital of the University of Pennsylvania (HUP), Penn Presbyerian Medical Center (PPMC), Pennsylvania Hospital (PAH), Chester County Hospital (CCH), and Penn Medicine Princeton Medical Center (PMC), and multiple outpatient practices and alliances, covering over 6.5 million unique patients and more than 50 million clinical encounters from the Greater Philadelphia Metropolitan area, Central PA, DE, and Southern NJ. The study protocol (Table S1) outlined eligibility criteria, treatment strategies, and outcomes for an ideal randomized trial, which we subsequently emulated using observational data. Adults aged ≥50 years with T2D who newly initiated a GLP‐1 RA, SGLT2is, or DPP4is were eligible for inclusion. Exclusions included prior dementia, end‐stage renal disease, or use of these agents in the preceding year. The index date was defined as the date of the first prescription. To ensure active engagement with the healthcare system and adequate capture of baseline diagnoses, medications, and clinical measurements, patients were required to have ≥1 healthcare encounter in the 12 months before the index date.

External validation was performed using de‐identified EHR data collected on 25 February 2025, in TriNetX US Collaborative Network from the TriNetX database, which is a global, federated health research network and provided access to EHRs from approximately 116 million patients from 69 healthcare organizations.^35^ This study followed the Strengthening the Reporting of Observational Studies in Epidemiology (STROBE) guidelines^36^ and was approved by the University of Pennsylvania Institutional Review Board (# 853466).

### 2.2 Exposures and outcomes

Exposures are defined as the first recorded prescription order for a GLP‐1 RA, DPP4i, or SGLT2i following a 12‐month washout period without prior use of any of these agents (Table S2). Consistent with an intention‐to‐treat design, no minimum duration of medication use was required, and follow‐up continued regardless of subsequent discontinuation, switching, or augmentation. The primary outcome was incident dementia, with Alzheimer's disease as a secondary outcome, identified by ICD‐10‐CM codes (Table S3). Follow‐up began at initiation and continued under an intention‐to‐treat approach until outcome, death, the patient's last recorded encounter in the health system, or the end of the study period.

### 2.3 Confounding factors

Baseline covariates were selected based on clinical relevance and existing literature.^26^ These covariates included sociodemographic characteristics (age, sex, EHR‐derived race/ethnicity, and health insurance), site index, care setting at cohort entry (emergency, inpatient, outpatient, or others), calendar year, and Charlson Comorbidity Index score at entry. We also considered healthcare utilization metrics (emergency, outpatient, and inpatient visits), key clinical measurements (e.g., vital signs, liver and kidney function, and lipid profiles), diabetes‐related complications (e.g., diabetic nephropathy, neuropathy, and retinopathy), comorbidities, and concomitant medications within 1 year before the index. Missing clinical measurements were imputed using multiple imputation by chained equations (MICE), implemented with predictive mean matching to preserve plausible clinical ranges.^37^

### 2.4 Statistical analyses

We conducted pairwise comparisons of GLP‐1 RAs vs. DPP4is and GLP‐1 RAs vs. SGLT2is. To reduce confounding, 1:1 PS matching was applied using logistic regression with a calliper of 0.05 standard deviations.^38^ Covariate balance was assessed using standardized mean differences (<0.1 indicating acceptable^39^). Cox proportional hazards models estimated hazard ratios (HRs) and 95% confidence intervals (CIs), with Kaplan–Meier curves for cumulative incidence.

Sensitivity analyses included IPTW to the unmatched cohort to adjust for confounding by weighting individuals based on the inverse of their predicted probability of receiving their actual treatment, Fine and Grey competing risk models to account for death as a competing risk,^40^ use of clinical dementia onset (1 year before recoded diagnosis) to account for the latency between symptom onset and diagnosis,^26^ sequential exclusion of patients with conditions that may confound dementia risk, and external validation in TriNetX with random‐effects meta‐analysis of pooled estimates.^41^, ^42^

We also considered the application of a more specific dementia definition requiring at least one dementia ICD‐10‐CM code after the index date and a prescription for a dementia‐specific medication within 12 months, with the event date defined as the date of the first post‐index dementia diagnosis (Table S4); incorporation of strong anticholinergic medications into the propensity‐score model to address potential confounding (Table S5); a fixed 2‐year follow‐up assessment in which all individuals were administratively censored at 24 months after treatment initiation to evaluate the robustness of effect estimates to differential follow‐up time; evaluation of vascular dementia and mixed/other dementia as alternative outcomes to explore subtype‐specific associations (Table S3). We additionally conducted negative control outcome analyses^43^ using deviated nasal septum, impacted cerumen, and regular astigmatism (Table S6) and calculated E‐value^44^ to assess the potential impact of unmeasured confounding.

Subgroup analyses were performed to explore potential effect modification in GLP‐1 RA‐associated dementia risk across key variables, including age (≥65 or <65 years), sex (female or male), race and ethnicity (non‐Hispanic White, non‐Hispanic Black, or others), baseline obesity (yes or no), hypertension (yes or no), heart failure (yes or no), cerebrovascular disease (yes or no), chronic kidney disease (yes or no), insulin use (yes or no), and GLP‐1 RA subtype (liraglutide, semaglutide, or others agents).

Analyses were conducted using R version 4.4.2, with statistical significance set at a two‐sided α of 0.05.

## 3 RESULTS

### 3.1 Study population

**FIGURE 1:**
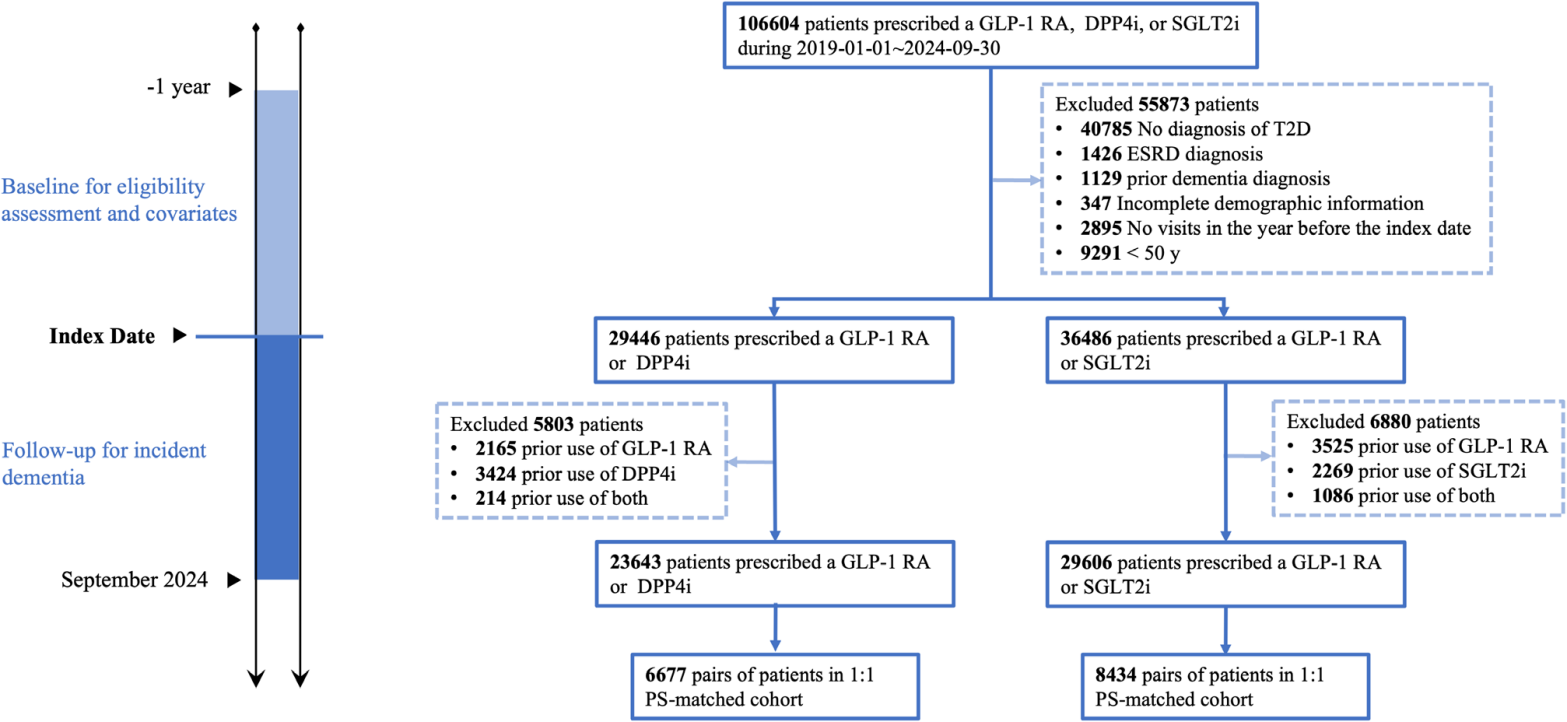
Target trial emulation design (left) and flow chart of patient selection (right) by using Penn Medicine EHR data. DPP4i, dipeptidyl peptidase‐4 inhibitor; ESRD, end‐stage renal disease; GLP‐1 RA, glucagon‐like peptide‐1 receptor agonist; HR, hazard ratio; IR, incidence rate; NHB, non‐Hispanic Black; NHW, non‐Hispanic White; PS, propensity score; SGLT2i, sodium–glucose cotransporter‐2 inhibitor; T2D, type 2 diabetes.

23 643 individuals were included in the GLP‐1 RA versus DPP4i cohort, and 29 606 in the GLP‐1 RA versus SGLT2i cohort. 6677 matched pairs were identified in the GLP‐1 RA versus DPP4i cohort (mean [SD] age, 64.7 [8.6] years for GLP‐1RA users; 65.7 [9.5] years for DPP4i users), and 8434 matched pairs in the GLP‐1 RA versus SGLT2i cohort (mean [SD] age, 64.1 [8.6] years for GLP‐1RA users; 64.4 [8.7] years for SGLT2i users). Post‐matching baseline characteristics were well‐balanced across all covariates (standardized mean differences <0.1) (Table 1). Pre‐matching baseline characteristics are provided in Table S7. Study design and cohort assembly using Penn Medicine EHR data are illustrated in Figure 1. Propensity score distributions before and after matching are shown in Figures S1 and S2. TriNetX cohort construction is presented in Tables S8 and S9. Reasons for censoring for both matched pairs can be found in Table S10.

**TABLE 1:** Baseline characteristics of patients initiating treatment with GLP‐1 RA versus DPP4i and SGLT2i after 1:1 PS matching using Penn Medicine EHR data.^a^

Characteristics	GLP‐1 RA vs. DPP4i (*n* = 6677 pairs)			GLP‐1 RA vs. SGLT2i (*n* = 84 34 pairs)		
	GLP‐1 RA	DPP4i	SMD^b^	GLP‐1 RA	SGLT2i	SMD^b^
Sex			0.004			0.022
Female	3239 (48.7)	3262 (48.9)		4146 (49.2)	4238 (50.2)	
Male	3428 (51.3)	3415 (51.1)	0.009	4288 (50.8)	4196 (49.8)	0.02
Mean age (SD), y	64.70 (8.61)	65.72 (9.46)	0.034	64.16 (8.55)	63.91 (8.60)	0.029
Age			0.019			0.023
<65 years	3290 (49.3)	3354 (50.2)		4533 (53.7)	4633 (54.2)	
≥65 years	3387 (50.7)	3323 (49.8)		3901 (46.3)	3806 (45.1)	
Race/ethnicity			0.022			0.019
Asian	288 (4.3)	276 (4.1)		292 (3.5)	270 (3.2)	
Hispanic	309 (4.6)	306 (4.6)		375 (4.4)	371 (4.4)	
Non‐Hispanic Black	1886 (28.2)	1933 (29.0)		2737 (32.5)	2709 (32.1)	
Non‐Hispanic White	3728 (55.8)	3721 (55.7)		4516 (53.5)	4578 (54.3)	
Other/Unknown	466 (7.0)	441 (6.6)		514 (6.1)	506 (6.0)	
Health insurance			0.022			0.024
Commercial	2463 (36.9)	2508 (37.6)		3314 (39.3)	3406 (40.4)	
Medicaid	550 (8.2)	570 (8.5)		829 (9.8)	813 (9.6)	
Medicare	3038 (45.5)	2966 (44.4)		3543 (42.0)	3499 (41.5)	
Self‐Pay/Other	626 (9.4)	633 (9.5)		748 (8.9)	716 (8.5)	
Site ID			0.023			0.010
1	1305 (19.5)	1296 (19.4)		1806 (21.4)	1788 (21.2)	
2	3097 (46.4)	3151 (47.2)		3983 (47.2)	3976 (47.1)	
3	778 (11.7)	782 (11.7)		1015 (12.0)	1013 (12.0)	
4	518 (7.8)	516 (7.7)		582 (6.9)	599 (7.1)	
5	835 (12.5)	792 (11.9)		881 (10.4)	887 (10.5)	
Other	144 (2.2)	140 (2.1)		167 (2.0)	171 (2.0)	
Patient setting			0.008			0.005
Emergency	38 (0.6)	36 (0.5)		44 (0.5)	47 (0.6)	
Inpatient	291 (4.4)	298 (4.5)		284 (3.4)	283 (3.4)	
Others	48 (0.7)	45 (0.7)		51 (0.6)	52 (0.6)	
Outpatient	6300 (94.4)	6298 (94.3)		8055 (95.5)	8052 (95.5)	
CCI			0.014			0.007
0	1103 (16.5)	1093 (16.4)		1049 (12.4)	1067 (12.7)	
1 ~ 2	453 (6.8)	476 (7.1)		665 (7.9)	669 (7.9)	
≥3	5121 (76.7)	5108 (76.5)		6720 (79.7)	6698 (79.4)	
Emergency visits			0.011			0.007
0	1054 (15.8)	1046 (15.7)		996 (11.8)	1014 (12.0)	
1 ~ 2	5251 (78.6)	5274 (79.0)		6931 (82.2)	6915 (82.0)	
≥3	372 (5.6)	357 (5.3)		507 (6.0)	505 (6.0)	
Inpatient visits			0.016			0.012
0	890 (13.3)	900 (13.5)		847 (10.0)	854 (10.1)	
1 ~ 2	4908 (73.5)	4933 (73.9)		6583 (78.1)	6609 (78.4)	
≥3	879 (13.2)	844 (12.6)		1004 (11.9)	971 (11.5)	
Outpatient visits			0.014			0.010
0	270 (4.0)	268 (4.0)		252 (3.0)	265 (3.1)	
1 ~ 2	580 (8.7)	606 (9.1)		638 (7.6)	649 (7.7)	
≥3	5827 (87.3)	5803 (86.9)		7544 (89.4)	7520 (89.2)	
Mean health examination results (SD)						
BMI, kg/m^2^	33.26 (7.52)	33.16 (8.47)	0.012	34.43 (7.75)	34.55 (8.41)	0.015
Systolic blood pressure, mmHg	132.55 (18.23)	132.51 (18.62)	0.002	132.59 (17.98)	132.72 (18.89)	0.007
Diastolic blood pressure, mmHg	76.94 (10.82)	76.94 (11.06)	0.001	77.21 (10.69)	77.45 (10.86)	0.022
HbA1c, %	8.68 (6.08)	8.70 (3.50)	0.004	8.51 (5.12)	8.48 (2.74)	0.008
Non‐fasting blood glucose, mg/dL	171.07 (83.27)	170.95 (72.31)	0.002	169.75 (81.32)	171.95 (77.54)	0.028
Alanine aminotransferase, IU/L	27.46 (41.80)	28.14 (58.38)	0.013	26.83 (41.49)	27.03 (45.41)	0.004
Aspartate aminotransferase, IU/L	25.42 (47.49)	26.06 (55.73)	0.012	25.58 (51.04)	25.48 (51.50)	0.002
Alkaline phosphatase, IU/L	83.06 (42.15)	83.06 (52.50)	<0.001	83.92 (48.36)	83.33 (40.13)	0.013
eGFR, mL/min/1.73 m^2^	74.69 (22.34)	75.06 (23.83)	0.016	75.46 (22.36)	75.94 (22.79)	0.021
Creatinine, mg/dL	1.07 (0.50)	1.06 (0.60)	0.002	1.06 (0.50)	1.05 (0.53)	0.002
Total cholesterol, mg/dL	161.55 (47.45)	161.72 (48.95)	0.004	160.80 (48.52)	161.74 (56.97)	0.018
Triglyceride, mg/dL	160.34 (97.20)	161.69 (104.72)	0.013	161.13 (103.36)	163.10 (112.27)	0.018
HD cholesterol L, mg/dL	46.50 (13.64)	46.39 (14.53)	0.008	46.68 (13.99)	46.68 (14.39)	<0.001
LDL cholesterol, mg/dL	93.46 (69.61)	94.63 (73.83)	0.016	94.45 (72.44)	95.91 (79.51)	0.019
Diabetes‐related conditions						
Diabetic nephropathy	1060 (15.9)	1009 (15.1)	0.021	1392 (16.5)	1387 (16.4)	0.002
Diabetic retinopathy	428 (6.4)	435 (6.5)	0.004	654 (7.8)	654 (7.8)	<0.001
Diabetic neuropathy	851 (12.7)	847 (12.7)	0.002	1250 (14.8)	1202 (14.3)	0.016
Diabetic peripheral vascular disease	366 (5.5)	387 (5.8)	0.014	495 (5.9)	504 (6.0)	0.005
Other specified complications	2862 (42.9)	2834 (42.4)	0.008	3878 (46.0)	3887 (46.1)	0.002
Other unspecified complications	665 (10.0)	640 (9.6)	0.013	984 (11.7)	976 (11.6)	0.003
Comorbid conditions						
Hypertension	5350 (80.1)	5328 (79.8)	0.008	6937 (82.3)	6830 (81.0)	0.033
Heart failure	864 (12.9)	832 (12.5)	0.014	1197 (14.2)	1137 (13.5)	0.021
Cerebrovascular disease	751 (11.2)	753 (11.3)	0.001	961 (11.4)	887 (10.5)	0.028
Obesity	2524 (37.8)	2550 (38.2)	0.008	3953 (46.9)	4055 (48.1)	0.024
Dyslipidemia	5161 (77.3)	5170 (77.4)	0.003	6683 (79.2)	6600 (78.3)	0.024
Chronic kidney disease	1219 (18.3)	1168 (17.5)	0.020	1540 (18.3)	1497 (17.7)	0.013
Asthma	823 (12.3)	806 (12.1)	0.008	1151 (13.6)	1169 (13.9)	0.006
COPD	541 (8.1)	518 (7.8)	0.013	705 (8.4)	666 (7.9)	0.017
Mild cognitive impairment	63 (0.9)	65 (1.0)	0.003	86 (1.0)	71 (0.8)	0.019
Parkinson's disease	36 (0.5)	41 (0.6)	0.010	43 (0.5)	34 (0.4)	0.016
Anxiety disorder	1249 (18.7)	1236 (18.5)	0.005	1769 (21.0)	1746 (20.7)	0.007
Mood disorder	1240 (18.6)	1224 (18.3)	0.006	1754 (20.8)	1743 (20.7)	0.003
Substance use disorder	830 (12.4)	806 (12.1)	0.011	1127 (13.4)	1094 (13.0)	0.012
Schizophrenia	49 (0.7)	51 (0.8)	0.003	56 (0.7)	62 (0.7)	0.009
Sleep disorder	1836 (27.5)	1800 (27.0)	0.012	2792 (33.1)	2843 (33.7)	0.013
Vitamin b12 deficiency	71 (1.1)	71 (1.1)	<0.001	114 (1.4)	114 (1.4)	<0.001
Rheumatoid arthritis	129 (1.9)	137 (2.1)	0.009	176 (2.1)	156 (1.8)	0.017
Concomitant drugs						
Antidepressants	160 (2.4)	98 (1.5)	0.067	286 (3.4)	197 (2.3)	0.063
Antipsychotics	54 (0.8)	28 (0.4)	0.050	90 (1.1)	63 (0.7)	0.034
ACEi/ARB	293 (4.4)	202 (3.0)	0.072	575 (6.8)	424 (5.0)	0.076
Beta blockers	134 (2.0)	93 (1.4)	0.048	279 (3.3)	205 (2.4)	0.053
Calcium‐channel blockers	176 (2.6)	103 (1.5)	0.076	333 (3.9)	241 (2.9)	0.060
Corticosteroids	133 (2.0)	78 (1.2)	0.066	224 (2.7)	159 (1.9)	0.052
Diuretics	244 (3.7)	176 (2.6)	0.058	540 (6.4)	400 (4.7)	0.072
Lipid‐lowering drugs	366 (5.5)	263 (3.9)	0.073	732 (8.7)	558 (6.6)	0.078
NSAIDs	132 (2.0)	68 (1.0)	0.079	271 (3.2)	179 (2.1)	0.068
Other antidiabetic drugs						
a‐Glucosidase inhibitors	11 (0.2)	11 (0.2)	<0.001	17 (0.2)	15 (0.2)	0.005
Insulin	1859 (27.8)	1825 (27.3)	0.011	2505 (29.7)	2494 (29.6)	0.003
Meglitinides	140 (2.1)	129 (1.9)	0.012	164 (1.9)	169 (2.0)	0.004
Metformin	2924 (43.8)	2887 (43.2)	0.011	4015 (47.6)	3962 (47.0)	0.013
Sulfonylureas	1228 (18.4)	1211 (18.1)	0.007	1508 (17.9)	1503 (17.8)	0.002
Thiazolidinediones	160 (2.4)	171 (2.6)	0.011	204 (2.4)	191 (2.3)	0.010
DPP4is	–	–	–	763 (9.0)	797 (9.4)	0.014
SGLT2is	618 (9.3)	605 (9.1)	0.007	–	–	–

Abbreviations: ACEi, angiotensin‐converting enzyme inhibitor; ARB, angiotensin‐receptor blocker; BMI, body mass index; CCI, Charlson Comorbidity Index; COPD, chronic obstructive pulmonary disease; DPP4i, dipeptidyl peptidase‐4 inhibitor; eGFR, estimated glomerular filtration rate; GLP‐1 RA, glucagon‐like peptide‐1 receptor agonist; HDL, high‐density lipoprotein; LDL, low‐density lipoprotein; NSAID, nonsteroidal anti‐inflammatory drug; PS, propensity score; SGLT2i, sodium–glucose cotransporter‐2 inhibitor.

^a^ Values are numbers (percentages) unless otherwise indicated.

^b^ After PS matching, an SMD ≤0.1 indicates a balance between the two groups.

### 3.2 Primary and secondary outcome analyses

**FIGURE 2:**
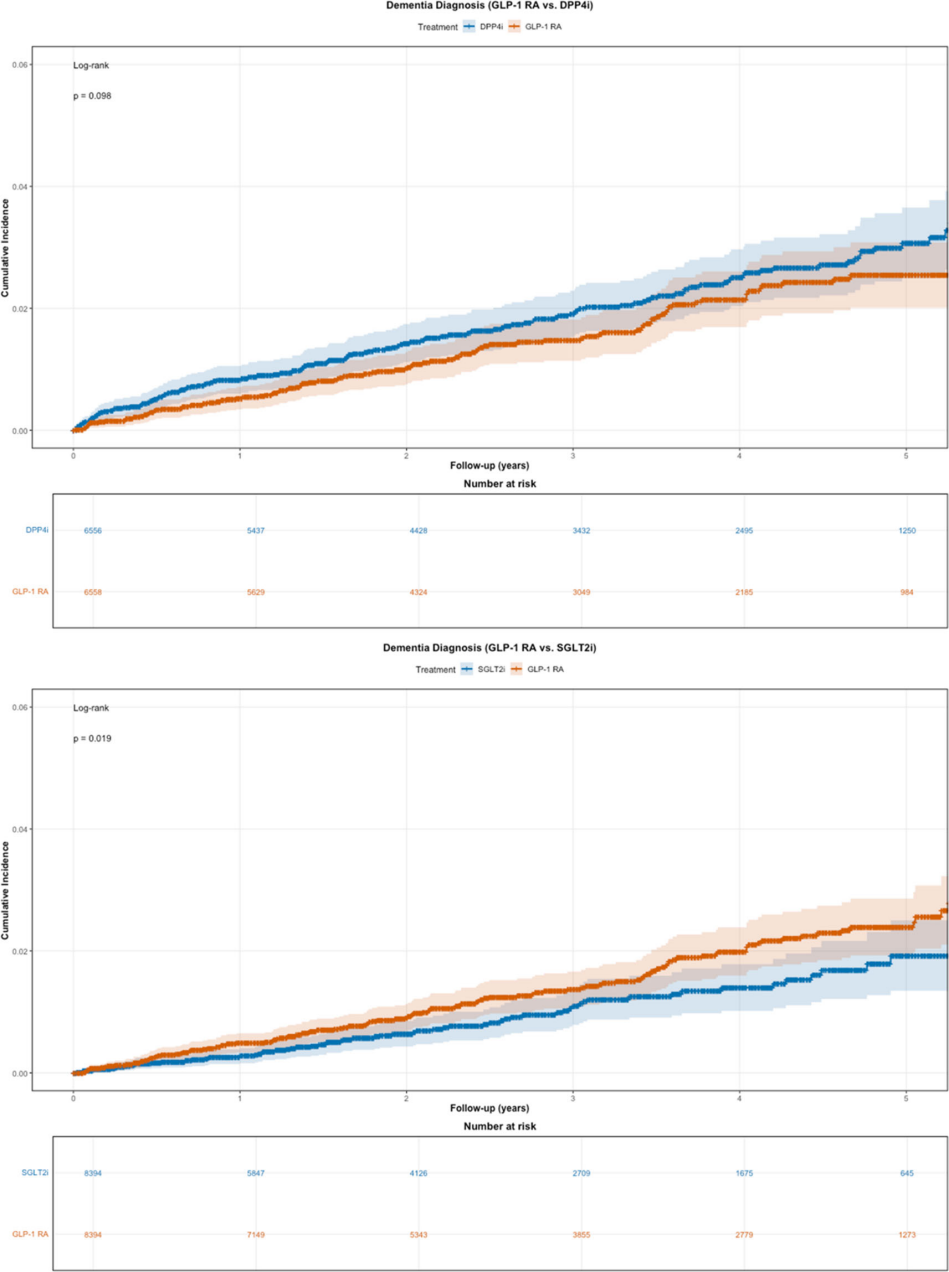
Cumulative incidence of dementia over time in the 1:1 PS‐matched cohorts for GLP‐1 RAs vs. DPP4is (top) and GLP‐1 RAs vs. SGLT2is (bottom) using Penn Medicine EHR data. The figure includes a risk table beneath a Kaplan–Meier curve, showing each cohort's number of patients at risk every year. DPP4i, dipeptidyl peptidase‐4 inhibitor; GLP‐1 RA, glucagon‐like peptide‐1 receptor agonist; PS, propensity score; SGLT2i, sodium–glucose cotransporter‐2 inhibitor.

As shown in Table 2, during follow‐up (median, 3.0 years [interquartile range, IQR, 1.6–4.7] for the DPP4i comparison; 2.4 years [IQR, 1.1–4.2] for the SGLT2i comparison), 109 dementia events occurred among GLP‐1 RA users compared with 148 in DPP4i users (5.49 vs. 7.26 per 1000 person‐years; HR, 0.76; 95% CI, 0.59–0.97). In contrast, 127 events occurred among GLP‐1 RA users versus 64 events in SGLT2i users (5.16 vs. 3.37 per 1000 person‐years; HR, 1.54; 95% CI, 1.13–2.07). Kaplan–Meier curves illustrating the cumulative incidence of dementia by treatment group for the matched groups are shown in Figure 2. Differences in Alzheimer's disease risk followed similar trends but were not statistically significant (Figure S3).

**TABLE 2:** Primary and secondary outcomes in 1:1 PS‐matched cohorts for GLP‐1 RAs versus DPP4is and SGLT2is using Penn Medicine EHR data.

Outcomes	GLP‐1 RA vs. DPP4i		GLP‐1 RA vs. SGLT2i	
	GLP‐1 RA	DPP4i	GLP‐1 RA	SGLT2i
*Primary outcome*				
Dementia diagnosis				
Events/Patients at risk, n/N	109/6677	148/6677	127/8434	64/8434
Median follow‐up (IQR), y	2.81 (1.61, 4.62)	3.20 (1.50, 4.75)	2.72 (1.47, 4.61)	1.93 (0.73, 3.59)
IR per 1000 person‐years	5.49	7.26	5.16	3.37
HR (95% CI)	0.76 (0.59, 0.97)	Reference	1.53 (1.13, 2.07)	Reference
*Secondary outcome*				
Alzheimer's disease				
Events/Patients at risk, n/N	9/6677	12/6677	10/8434	6/8434
Median follow‐up (IQR), y	2.85 (1.64, 4.63)	3.27 (1.54, 4.80)	2.76 (1.49, 4.62)	1.95 (0.74, 3.60)
IR per 1000 person‐years	0.45	0.58	0.40	0.32
HR (95% CI)	0.77 (0.32, 1.82)	Reference	1.24 (0.45, 3.42)	Reference

Abbreviations: DPP4i, dipeptidyl peptidase‐4 inhibitor; GLP‐1 RA, glucagon‐like peptide‐1 receptor agonist; HR, hazard ratio; IR, incidence rate; PS, propensity score; SGLT2i, sodium–glucose cotransporter‐2 inhibitor.

### 3.3 Sensitivity analyses

**FIGURE 3:**
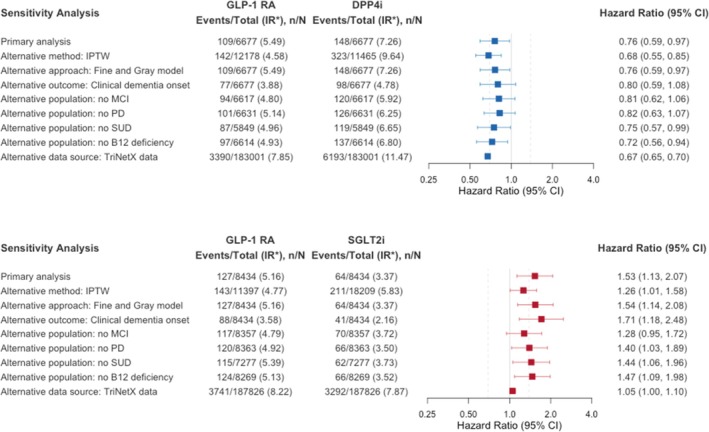
Sensitivity analyses for dementia diagnosis in the 1:1 PS‐matched cohorts for GLP‐1 RAs vs. DPP4is (top) and GLP‐1 RAs vs. SGLT2is (bottom) using Penn Medicine EHR data. DPP4i, dipeptidyl peptidase‐4 inhibitor; GLP‐1 RA, glucagon‐like peptide‐1 receptor agonist; IPTW, inverse probability of treatment weighting; MCI, mild cognitive impairment; PD, Parkinson's disease; PS, propensity score; SGLT2i, sodium–glucose cotransporter‐2 inhibitor; SUD, substance use disorder.

Findings were consistent across multiple sensitivity analyses (Figure 3). IPTW models produced results like the primary analysis (vs. DPP4i: HR, 0.76 [95% CI, 0.59–0.97]; vs. SGLT2i: HR, 1.53 [95% CI, 1.13–2.07]), as did competing risk models accounting for death (vs. DPP4i: HR, 0.68 [95% CI, 0.55–0.85]; vs. SGLT2i: HR, 1.53 [95% CI, 1.13–2.07]). When dementia onset was defined 1 year before recorded diagnosis, GLP‐1 RA use was associated with a nonsignificant lower risk versus DPP4i (77 vs. 98 events; HR, 0.80 [95% CI, 0.59–1.08]) and a higher risk versus SGLT2i (88 vs. 41 events; HR, 1.71 [95% CI, 1.18–2.48]). Excluding patients with baseline mild cognitive impairment, Parkinson's disease, substance use disorder, or vitamin B12 deficiency did not materially alter findings.

External validation using TriNetX confirmed the protective association with DPP4i (HR, 0.67 [95% CI, 0.65–0.70]) but showed no difference with SGLT2i (HR, 1.05 [95% CI, 1.00–1.10]). Pooled analyses across Penn Medicine and TriNetX reinforced these results (vs. DPP4i: HR, 0.68 [95% CI, 0.65–0.70]; vs. SGLT2i: HR, 1.23 [95% CI, 0.85–1.77]) (Figure S4). For Alzheimer's disease, pooled HRs were 0.69 (95% CI, 0.64–0.75) versus DPP4i and 1.14 (95% CI, 1.03–1.25) versus SGLT2i (Figure S5).

Results were directionally consistent with the primary analysis when applying a stricter dementia definition that required at least one post‐index diagnosis and a dementia‐specific medication within 12 months (Table S11), when incorporating strong anticholinergic medications into the propensity‐score model (Table S12), and when using a fixed 2‐year follow‐up window in which all participants were administratively censored at 24 months (Table S13). Findings were also consistent when vascular dementia and mixed/other dementia were evaluated as alternative outcomes, although estimates for vascular dementia were imprecise due to limited events (Table S14). Negative control outcome analyses showed no significant associations, suggesting the absence of residual bias (Table S15). The E‐value for the GLP‐1 RA versus DPP4i comparison was 1.96, and for the GLP‐1 RA versus SGLT2i comparison was 2.43, indicating that an unmeasured confounder would need to be associated with both treatment assignment and dementia risk by hazard ratios of at least this magnitude to fully explain the observed associations.

### 3.4 Subgroup analyses

Subgroup analyses by age, sex, race/ethnicity, obesity, hypertension, heart failure (HF), cerebrovascular disease, chronic kidney disease, insulin use, and GLP‐1 RA subtype showed consistent results. Interaction tests indicated no significant effect modification (*p* > 0.05 for all interactions) (Figure S6). In obesity‐stratified analyses, GLP‐1 RA use was associated with significantly lower dementia risk among patients without obesity versus DPP4i (HR, 0.64; 95% CI, 0.47–0.89) and a nonsignificant difference versus SGLT2i (HR, 1.25; 95% CI, 0.85–1.82). Among individuals with obesity, no significant difference was observed versus DPP4i (HR, 0.99; 95% CI, 0.65–1.50), but dementia risk was higher versus SGLT2i (HR, 2.11; 95% CI, 1.27–3.51).

By subtype, semaglutide was associated with lower dementia risk versus DPP4i (HR, 0.65 [95% CI, 0.42–0.99]) but not versus SGLT2i (HR, 1.26 [95% CI, 0.75–2.12]); liraglutide showed no significant differences (vs DPP4i: HR, 0.89 [95% CI, 0.51–1.55]; vs SGLT2i: HR, 1.17 [95% CI, 0.64–2.14]).

## 4 DISCUSSION

In this target trial emulation study of older adults with T2D, GLP‐1 RA use was associated with lower dementia risk versus DPP4i, but higher risk versus SGLT2i. These associations remained directionally consistent across multiple sensitivity analyses and were further supported by external validation in TriNetX, strengthening confidence in the robustness of the findings. Although no statistically significant effect modification was observed across subgroups, the observed patterns suggest clinically meaningful hypotheses that warrant investigation in future studies.

The observed protective association of GLP‐1 RAs versus DPP4is aligns with the known neurobiological actions of GLP‐1 signalling, including reduced neuroinflammation, enhanced synaptic plasticity, and improved central insulin sensitivity, key processes implicated in neurodegenerative disease pathophysiology.^13^ Preclinical studies also suggest that GLP‐1 RAs may cross the blood–brain barrier and directly activate GLP‐1 receptors.^45^ Although DPP4is prolong endogenous GLP‐1 activity by inhibiting the DPP4 enzyme,^46^ this indirect mechanism is less potent than direct receptor agonism.^47^ Our findings are consistent with several observational studies,^24^, ^25^ but differ from a recent Medicare claims‐based analysis^32^ that reported no overall difference in dementia risk between GLP‐1 RA and DPP4i users. These differences likely reflect variations in data sources, covariate capture (including adiposity measures), population characteristics, and secular prescribing patterns rather than true contradiction, and the studies should be viewed as complementary. Taken together, our findings suggest that GLP‐1 RAs may confer cognitive benefits relative to DPP4is; however, whether this reflects a true neuroprotective effect of GLP‐1 RAs or comparatively weaker cognitive effects of DPP4is cannot be fully distinguished in an observational design and warrants investigation in future mechanistic or randomized studies.

The finding that SGLT2i use was associated with a lower dementia risk than GLP‐1 RAs adds to emerging evidence suggesting potential cognitive benefits of SGLT2is. Although SGLT2is are primarily known for their cardiovascular benefits, experimental data suggest possible cognitive mechanisms, including reduced neuroinflammation and improved cerebral perfusion.^20^, ^48^ Given the mixed findings in prior observational and trial‐based studies.^25^, ^26^, ^27^, ^28^, ^29^, ^30^, ^33^ we must interpret these results cautiously. Some observational studies reported lower dementia risk with SGLT2i versus GLP‐1 RA,^27^, ^28^ while others found no significant difference^26^, ^30^; together, the evidence remains inconclusive and highlights the need for head‐to‐head randomized trials with cognitive outcomes; together, the evidence remains inconclusive and highlights the need for head‐to‐head trials with cognitive endpoints. Although the Penn Medicine estimate suggested a modestly higher dementia risk for GLP‐1 RAs versus SGLT2is and the TriNetX estimate was closer to null, the direction of association was similar across both datasets, with differences in magnitude likely reflecting underlying variations in population characteristics, prescribing patterns, and data structures rather than a fundamentally discordant effect.

Sensitivity analyses further supported the robustness of the findings. Across multiple approaches, including IPTW in the unmatched cohort, Fine‐Grey competing risk models, application of a clinically informed dementia onset definition, sequential exclusion of comorbid conditions, use of vascular and mixed/other dementia as alternative outcomes, incorporation of a stricter dementia definition requiring both a diagnosis code and a dementia‐specific medication, adjustment for anticholinergic burden, and a fixed 2‐year follow‐up, the direction of associations remained consistent with the primary analysis, although estimates were imprecise when case counts were low. Negative control outcome analyses did not indicate residual bias, and E‐values suggested that a substantial unmeasured confounder would be required to fully explain the observed treatment‐dementia associations. External validation in TriNetX, together with pooled random‐effects estimates, further reinforced the overall stability of the findings.

Specifically, excluding patients with baseline MCI or other related neurologic conditions did not materially alter the observed associations. This is particularly important given that medication effects may vary by stages of cognitive impairment.^48^, ^49^ Similar patterns were observed when excluding patients with Parkinson's disease, substance use disorder, or vitamin B12 deficiency, indicating that secondary or reversible causes of cognitive impairment did not drive the observed associations. Parkinson's disease was included as a baseline covariate, and although biologically relevant for GLP‐1 RAs, incident Parkinson's cases were too few to allow meaningful secondary analysis. Future work with larger datasets and longer follow‐up periods will be necessary to fully evaluate this outcome. Taken together, these multiple sensitivity analyses reinforce the stability of the primary findings despite potential limitations in outcome misclassification, confounding, and follow‐up variation.

All subgroup analyses were exploratory, not adjusted for multiple comparisons, and did not demonstrate statistically significant effect modification; therefore, numerical differences should be interpreted with caution. Although some variation was observed across racial/ethnic groups, cardiometabolic risk strata, and GLP‐1 RA subtypes, these patterns were not statistically significant and may reflect residual confounding, differential diagnosis, or coding practices, treatment selection, or limited statistical power rather than true biological heterogeneity. For example, the protective association of GLP‐1 RAs versus DPP4is appeared somewhat more pronounced among non‐Hispanic White individuals, while higher dementia risk for GLP‐1 RAs versus SGLT2is appeared in both non‐Hispanic White and non‐Hispanic Black individuals, but none of these differences were significant and should be considered hypothesis‐generating only. Collectively, these exploratory findings highlight potential areas for future research in larger and more diverse cohorts with longer follow‐up to clarify whether such numerical differences reflect biological variation or social and structural determinants of health. Subgroup analyses were not repeated in TriNetX, as this external dataset was used solely to validate overall effect estimates, and its multisite structure may limit the reliability of subgroup‐level comparisons.

Our study has several strengths. First, we employed a target trial emulation framework, which explicitly aligned eligibility criteria, treatment strategies, follow‐up, and analytic approaches with a hypothetical randomized clinical trial, thereby enhancing causal inference from observational data. Second, the use of rich electronic health record data enabled the comprehensive capture of clinical covariates, including laboratory measurements such as haemoglobin A1c, allowing for more detailed adjustment than is feasible in many claims‐based studies.^24^, ^26^ Rigorous PS matching produced well‐balanced baseline characteristics between treatment groups, and the inclusion of two active comparators (DPP4is and SGLT2is) minimized confounding by indication and provided a nuanced assessment of the relative cognitive effects of GLP‐1 RAs in a real‐world setting. Third, the robustness of the findings was supported by an extensive set of sensitivity analyses, including IPTW using the full cohort, competing risk models, alternative dementia definitions, exclusion of individuals with conditions that may influence cognitive trajectories, adjustment for strong anticholinergic medications, fixed 2‐year follow‐up, and examination of dementia subtypes. External validation using TriNetX further strengthened generalizability and reproducibility.^24^, ^26^ Finally, to address the possibility of reverse causation, we incorporated a clinically informed onset window and accounted for mild cognitive impairment through both adjustment and exclusion, with results remaining consistent across analyses, although some residual bias cannot be completely excluded.

## 5 LIMITATIONS

Nonetheless, several limitations should be considered. Despite extensive adjustment, residual confounding from unmeasured variables, such as diabetes duration, frailty, genetic predisposition, environmental, and socioeconomic factors, may remain and could influence the observed associations.^3^, ^50^, ^51^, ^52^ Residual confounding by adiposity is also possible because BMI does not capture body composition or fat distribution, and adiposity changes relate differently to dementia risk across obesity strata.^53^ Because EHRs record prescription orders rather than dispensing, refills, or adherence, true medication duration could not be ascertained; exposure was therefore defined at first prescription, and discontinuation or treatment switching cannot be reliably captured, precluding per‐protocol or as‐treated analyses and leaving open the possibility of residual bias related to treatment changes.

EHR systems also lack formal enrolment structures, and although required ≥1 encounter in the prior year to improve covariate completeness, continuous observation cannot be guaranteed. The median follow‐up of 2.4–3.0 years is relatively short for dementia onset and may lead to under‐ascertainment; however, limited event counts made it infeasible to implement a 1‐year lagged follow‐up period that excludes early post‐initiation diagnoses. Dementia diagnoses in EHRs are prone to under‐detection and misclassification,^54^ and Alzheimer's disease is often coded as unspecified dementia, likely explaining the lower proportion of AD diagnoses in our cohort than epidemiological estimates.

Generalizability may be limited to academic and U.S.‐based healthcare settings. Differences in effect magnitude across Penn Medicine and TriNetX highlight the need for replication in diverse populations. Finally, PS matching improved internal validity but reduced the analytic sample; however, IPTW analyses retaining the full cohort yielded directionally consistent results, supporting the robustness of the findings.

## 6 CONCLUSIONS

In this large real‐world cohort of older adults with T2D, initiation of GLP‐1 RA use was associated with lower dementia risk versus DPP4i but higher risk versus SGLT2i. Results were consistent across extensive sensitivity analyses and showed consistent directional patterns across subgroups. Together, the findings suggest potential cognitive benefits of GLP‐1 RAs relative to DPP4is while underscoring the emerging neuroprotective profile of SGLT2is. Prospective, biomarker‐informed studies are needed to clarify underlying mechanisms and inform treatment decisions aimed at reducing dementia risk in diabetes populations.

## AUTHOR CONTRIBUTIONS

Authorship was determined using ICMJE recommendations. Ting Zhou and Yong Chen (University of Pennsylvania) contributed to the concept and design; Yong Chen (University of Pennsylvania) provided datasets for data analysis; Ting Zhou and Huilin Tang performed the statistical analysis; Ting Zhou visualized the analysis results; Ting Zhou, Huilin Tang, Bingyu Zhang, Dazheng Zhang, Yiwen Lu, Lu Li, Jiajie Chen, Yong Chen (Pfizer, New York City, NY, USA), David A. Asch, and Yong Chen (University of Pennsylvania) interpreted the results. Ting Zhou wrote the initial draft of the manuscript, and Yong Chen (University of Pennsylvania) supervised the study. All authors revised and approved the final version of the manuscript.

## FUNDING INFORMATION

This study was supported in part by the U.S. National Institutes of Health (U01TR003709, U24MH136069, RF1AG077820, R01AG073435, R56AG074604, R01LM013519, R01DK128237, R21AI167418, R21EY034179).

## CONFLICT OF INTEREST STATEMENT

Dr. David Asch is a partner and part‐owner of VAL Health and serves on the advisory boards of Thrive Global and Morpheus. Dr. Yong Chen (University of Pennsylvania) reported receiving personal fees from Merck & Co., Inc. and Pfizer Inc. outside the submitted work. Dr. Yong Chen (Pfizer, New York City, NY, USA) is a Pfizer employee and owns Pfizer stock. No potential conflicts of interest relevant to this article were reported by Ting Zhou, Huilin Tang, Bingyu Zhang, Dazheng Zhang, Yiwen Lu, Lu Li, and Jiajie Chen.

## Supporting information


**DATA S1.** Supplementary information.

## Data Availability

The detailed individual‐level patient data were extracted from the University of Pennsylvania Health System's electronic health records (EHRs). Due to the high risk of reidentification based on the number of unique patterns in the data, patient privacy regulations prohibit us from releasing the data publicly. The TriNetX data were collected and analyzed within the TriNetX Analytics platform. The TriNetX data request for the data access can be submitted via https://trinetx.com/.
