# Supplementary material for: Association between glucagon‐like peptide‐1 receptor agonists and risk of dementia in older adults with type 2 diabetes: A target trial emulation
Source: Diabetes Obes Metab. 2025 Dec 22;28(3):1984–96. doi: 10.1111/dom.70384 (PMC12890729; doi:10.1111/dom.70384)
Supplement: Supplementary file 1 — DATA S1. Supplementary information. [file DOM-28-1984-s001.docx]

**Supplementary Appendix**

**Zhou T, Huilin T, Zhang BY, et al. Association between Glucagon-Like Peptide-1 Receptor Agonists and Risk of Dementia in Older Adults with Type 2 Diabetes: A Target Trial Emulation.**

Table of Contents

[Table S1. Specification and emulation of pragmatic target trials 2](#_Toc215757489)

[Table S2. Exposures of interest and comparisons 3](#_Toc215757490)

[Table S3. Definitions of outcome, inclusion, and exclusion criteria, and baseline covariates 4](#_Toc215757491)

[Table S4. Antidementia drugs with ATC codes 5](#_Toc215757492)

[Table S5. Anticholinergic cognitive burden (ACB) level 3 medications with ATC codes 6](#_Toc215757493)

[Table S6. Negative control outcomes used in the study 7](#_Toc215757494)

[Table S7. Baseline characteristics of patients initiating treatment with GLP-1 RA versus DPP4i and SGLT2i before 1:1 PS matching using Penn Medicine EHR data* 8](#_Toc215757495)

[Figure S1. Propensity score density for GLP-1 RA (Treatment=1) versus DPP4i (Treatment=0) before and after 1:1 PS matching using Penn Medicine EHR data for primary analysis 10](#_Toc215757496)

[Figure S2. Propensity score density for GLP-1 RA (Treatment=1) versus SGLT2i (Treatment=0) before and after 1:1 PS matching using Penn Medicine EHR data for primary analysis 11](#_Toc215757497)

[Table S8. Details of cohort construction for GLP-1 RA vs DPP4i cohorts in TriNetX 12](#_Toc215757498)

[Table S9. Details of cohort construction for GLP-1 RA vs SGLT2i cohorts in TriNetX 13](#_Toc215757499)

[Table S10. Reasons for censoring in 1:1 PS-matched cohorts for GLP-1 RAs versus DPP4is and SGLT2is using Penn Medicine EHR data, N (%) ^*^ 14](#_Toc215757500)

[Figure S3. Cumulative incidence of Alzheimer’s disease over time in the 1:1 PS-matched cohorts for GLP-1 RAs vs. DPP4is (top) and GLP-1 RAs vs. SGLT2is (bottom) using Penn Medicine EHR data. The figure includes a risk table beneath a Kaplan-Meier curve, showing each cohort's number of patients at risk every year. 15](#_Toc215757501)

[Figure S4. Pooled estimate of hazard ratios for dementia diagnosis in the 1:1 PS-matched cohorts for GLP-1 RAs vs. DPP4is (top) and GLP-1 RAs vs. SGLT2is (bottom) using data from Penn Medicine and TriNetX. 16](#_Toc215757502)

[Figure S5. Pooled estimate of hazard ratios of Alzheimer’s disease diagnosis in the 1:1 PS-matched cohorts for GLP-1 RAs vs. DPP4is (top) and GLP-1 RAs vs. SGLT2is (bottom) using data from Penn Medicine and TriNetX. 17](#_Toc215757503)

[Table S11. Sensitivity analysis using a stricter dementia outcome in 1:1 PS-matched cohorts for GLP-1 RAs versus DPP4is and SGLT2is using Penn Medicine EHR data^*^ 18](#_Toc215757504)

[Table S12. Sensitivity analysis adjusting baseline strong anticholinergic medications in 1:1 PS-matched cohorts for GLP-1 RAs versus DPP4is and SGLT2is using Penn Medicine EHR data^*^ 19](#_Toc215757505)

[Table S13. Sensitivity analysis using a fixed 2-year follow-up window in 1:1 PS-matched cohorts for GLP-1 RAs versus DPP4is and SGLT2is using Penn Medicine EHR data^*^ 20](#_Toc215757506)

[Table S14. Sensitivity analysis for other dementia subtypes in 1:1 PS-matched cohorts for GLP-1 RAs versus DPP4is and SGLT2is using Penn Medicine EHR data^*^ 21](#_Toc215757507)

[Table S15. Negative control outcomes analysis in 1:1 PS-matched cohorts for GLP-1 RAs versus DPP4is and SGLT2is using Penn Medicine EHR data^*^ 22](#_Toc215757508)

[Figure S6. Subgroup analyses for dementia diagnosis in the 1:1 PS-matched cohorts for GLP-1 RAs vs. DPP4is (top) and GLP-1 RAs vs. SGLT2is (bottom) using Penn Medicine EHR data 23](#_Toc215757509)

**Table S1**. Specification and emulation of pragmatic target trials

| Approaches | Target Trial | Target Trial Emulation |
| --- | --- | --- |
| Eligibility criteria | Target trial among individuals:  • Age≥ 50 years between January 1, 2019 and September 30, 2024  • Type 2 diabetes diagnosis  • No study drug contraindication, i.e., end-stage renal disease  • No prescription for GLP-1RA, SGLT2is, or DPP4is within past year  • No history of dementia | Same as for the target trial |
| Treatment strategies | • For GLP-1RA vs. SGLT2i cohort: Initiation of GLP-1RA at baseline vs. Initiation of an SGLT2i at baseline.  • For GLP-1RA vs. DPP4i cohort: Initiation of GLP-1RA at baseline vs. Initiation of a DPP4i at baseline. | Same as for the target trial |
| Treatment assignment | Individuals are randomly assigned to a strategy at baseline. Individuals and their treating physicians will be aware of the assigned treatment strategy. | Individuals are assigned to  the strategy compatible with  their first prescription and  assumed randomization by  propensity-score matching  for baseline covariates. |
| Outcomes | Primary outcome: Diagnosis of dementia  Secondary outcome: Diagnosis of Alzheimer's disease | Same as for the target trial |
| Follow-up | For each eligible individual, follow-up starts at treatment assignment and ends on the occurrence of an outcome occurrence, death, or the end of the study period (September 30, 2024), whichever comes first. | Same as for the target trial |
| Causal contrasts | Intention-to-treat effect | Observational analogs of the intention-to-treat |
| Statistical analysis | Intention-to-treat analysis; Kaplan-Meier plot to show the cumulative incidence over time for each treatment strategy; Cox proportional hazards regression model to estimate hazard ratios    Sensitivity analyses: 1) using an alternative method, i.e., inverse probability of treatment weighting to adjust for confounding; 2) using an alternative approach, i.e., the Fine and Gray model to address the competing risk of death; 3) using an alternative outcome, i.e., clinical onset of dementia, defined as the year before the date of dementia diagnosis, assuming that the time between the onset of dementia and diagnosis was 1 year; 4) excluding participants with mild cognitive impairment (MCI) at baseline; 5) excluding participants with Parkinson's disease (PD) at baseline; 6) excluding participants with substance use disorder (SUD) at baseline; 7) excluding participants with vitamin B12 deficiency at baseline; 8) Using alternative data source, TriNetX data; 9) Conducting meta-analysis for EHR data from Penn Medicine and TriNetX system..    Subgroup analyses by 1) age (≥ 65 years vs. < 65 years); 2) sex (female vs. male); 3) race/ethnicity (non-Hispanic White vs. non-Hispanic Black vs. others except non-Hispanic White and non-Hispanic Black); 4) having a diagnosis of obesity at baseline (yes vs. no); 5) having a diagnosis of hypertension at baseline (yes vs. no); 6) having a diagnosis of heart failure at baseline (yes vs. no); 7) having a diagnosis of cerebrovascular disease at baseline (yes vs. no); 8) having a diagnosis of chronic kidney disease at baseline (yes vs.no); 9) use of insulin at baseline (yes vs. no); 10) molecular structure of GLP-1 RA (liraglutide vs. semaglutide vs. others except liraglutide and semaglutide. | Same as for the target trial |

Abbreviations: DPP4i:dipeptidyl peptidase-4 inhibitor; GLP-1 RA: glucagon-like peptide-1 receptor agonist; SGLT2i: sodium-glucose cotransporter-2 inhibitor.

**Table S2**. Exposures of interest and comparisons

| **Category** | **Individual drugs (ATC code)** |
| --- | --- |
| GLP-1RAs | Albiglutide (A10BJ04), Dulaglutide (A10BJ05), Exenatide (A10BJ01), Liraglutide (A10BJ02), Lixisenatide (A10BJ03), Semaglutide (A10BJ06), Tirzepatide (A10BK04) |
| DPP4i | Alogliptin (A10BH05), Linagliptin (A10BH04), Saxagliptin (A10BH03), Sitagliptin (A10BH01) |
| SGLT2i | Bexagliflozin (A10BK05), Canagliflozin (A10BK02), Dapagliflozin (A10BK01), Empagliflozin (A10BK03), Ertugliflozin (A10BK04), Sotagliflozin (A10BK06) |

Abbreviations: ATC: anatomical therapeutic chemical; DPP4i:dipeptidyl peptidase-4 inhibitor; GLP-1 RA: glucagon-like peptide-1 receptor agonist; SGLT2i: sodium-glucose cotransporter-2 inhibitor.

**Table S3**. Definitions of outcome, inclusion, and exclusion criteria, and baseline covariates

| **Variable** | **Definitions** |
| --- | --- |
| **Outcomes** |  |
| Dementia | ICD-10-CM codes: F01, F02, F03, G30 |
| Alzheimer’s disease | ICD-10-CM codes: G30 |
| Vascular dementia | ICD-10-CM codes: F01 |
| Mixed/other dementia | ICD-10-CM codes: F02, F03 |
| **Inclusion and exclusion criteria** |  |
| Type 2 diabetes mellitus | ICD-10-CM codes: E11 |
| End-stage renal disease | ICD-10-CM codes: N18.6 |
| Dementia | ICD-10-CM codes: F01, F02, F03, G30 |
| **Diabetes-related conditions** |  |
| Diabetic nephropathy | ICD-10-CM codes: E11.2 |
| Diabetic retinopathy | ICD-10-CM codes: E11.3 |
| Diabetic neuropathy | ICD-10-CM codes: E11.4 |
| Diabetic peripheral vascular disease | ICD-10-CM codes: E11.5 |
| Type 2 diabetes mellitus with other specified complications | ICD-10-CM codes: E11.6 |
| Type 2 diabetes mellitus with unspecified complications | ICD-10-CM codes: E11.8 |
| **Comorbid conditions** |  |
| Hypertension | ICD-10-CM codes: I10, I11, I12, I13, I15 |
| Heart failure | ICD-10-CM codes: I50 |
| Cerebrovascular diseases | ICD-10-CM codes: I60, I61, I62, I63, I65, I66, I67, I68, I69 |
| Obesity | ICD-10-CM codes: E66.9, E66.0, E66.2, E66.8, Z68.3, Z68.4 |
| Dyslipidemia | ICD-10-CM codes: E78 |
| Chronic kidney disease | ICD-10-CM codes: N18 |
| Asthma | ICD-10-CM codes: J45 |
| Chronic obstructive pulmonary disease | ICD-10-CM codes: J44 |
| Mild cognitive impairment | ICD-10-CM codes: G31.84 |
| Parkinson’s disease | ICD-10-CM codes: G20 |
| Anxiety disorders | ICD-10-CM codes: F40-F48 (F40, F41, F42, F43, F44, F45, F48) |
| Mood disorders | ICD-10-CM codes: F30-F39 (F30, F31, F32, F33, F34, F39) |
| Substance use disorders | ICD-10 codes: F10- F19 (F10, F11, F12, F13, F14, F15, F16, F17, F18, F19) |
| Schizophrenia | ICD-10-CM codes: F20 |
| Sleep disorder | ICD-10-CM codes: G47 |
| Vitamin b12 deficiency | ICD-10-CM codes: D51 |
| Rheumatoid arthritis | ICD-10-CM codes: M05, M06 |
| **Concomitant drugs** |  |
| Antidepressants | ATC codes: N06A |
| Antipsychotics | ATC codes: N05A |
| ACEi/ARB | ATC codes: C09 |
| Beta-blockers | ATC codes: C07 |
| Calcium channel blockers | ATC codes: C08 |
| Corticosteroids | ATC codes: A07EA |
| Diuretics | ATC codes: C03 |
| Lipid lowering drugs | ATC codes: C10 |
| NSAIDs | ATC codes: M01A |
| Other antidiabetic drugs | ATC codes:  Sulfonylureas (A10BB), Alpha-glucosidase inhibitors (A10BF), Thiazolidinediones (A10BG), insulin (A10A) and DPP4i (A10BH) or SGLT2i (A10BK) |

Abbreviations: ATC: anatomical therapeutic chemical; ICD-10-CM: International Classification of Diseases System, 10^th^ Revision, Clinical Modification.

**Table S4**. Antidementia drugs with ATC codes

| Drug Name | Drug Class | ATC Code |
| --- | --- | --- |
| Donepezil | Cholinesterase inhibitor | N06DA02 |
| Rivastigmine | Cholinesterase inhibitor | N06DA03 |
| Galantamine | Cholinesterase inhibitor | N06DA04 |
| Memantine | NMDA receptor antagonist | N06DX01 |
| Aducanumab | Anti-amyloid monoclonal antibody | N06DX02 |
| Lecanemab | Anti-amyloid monoclonal antibody | N06DX03 |
| Donanemab | Anti-amyloid monoclonal antibody | N06DX04 |
| Donepezil | Cholinesterase inhibitor | N06DA02 |
| Rivastigmine | Cholinesterase inhibitor | N06DA03 |
| Galantamine | Cholinesterase inhibitor | N06DA04 |
| Memantine | NMDA receptor antagonist | N06DX01 |
| Aducanumab | Anti-amyloid monoclonal antibody | N06DX02 |
| Lecanemab | Anti-amyloid monoclonal antibody | N06DX03 |
| Donanemab | Anti-amyloid monoclonal antibody | N06DX04 |
| Donepezil | Cholinesterase inhibitor | N06DA02 |
| Rivastigmine | Cholinesterase inhibitor | N06DA03 |
| Galantamine | Cholinesterase inhibitor | N06DA04 |
| Memantine | NMDA receptor antagonist | N06DX01 |
| Aducanumab | Anti-amyloid monoclonal antibody | N06DX02 |
| Lecanemab | Anti-amyloid monoclonal antibody | N06DX03 |
| Donanemab | Anti-amyloid monoclonal antibody | N06DX04 |
| Donepezil | Cholinesterase inhibitor | N06DA02 |
| Rivastigmine | Cholinesterase inhibitor | N06DA03 |
| Galantamine | Cholinesterase inhibitor | N06DA04 |
| Memantine | NMDA receptor antagonist | N06DX01 |
| Aducanumab | Anti-amyloid monoclonal antibody | N06DX02 |
| Lecanemab | Anti-amyloid monoclonal antibody | N06DX03 |
| Donanemab | Anti-amyloid monoclonal antibody | N06DX04 |
| Donepezil | Cholinesterase inhibitor | N06DA02 |

Abbreviations: ATC: anatomical therapeutic chemical; NMDA: N-methyl-D-aspartate.

**Table S5**. Anticholinergic cognitive burden (ACB) level 3 medications with ATC codes

| Drug Name | Drug Class | ATC Code |
| --- | --- | --- |
| Amitriptyline | Tricyclic antidepressant | N06AA09 |
| Clomipramine | Tricyclic antidepressant | N06AA04 |
| Imipramine | Tricyclic antidepressant | N06AA02 |
| Nortriptyline | Tricyclic antidepressant | N06AA10 |
| Doxepin | Tricyclic antidepressant | N06AA12 |
| Paroxetine | SSRI with strong anticholinergic effect | N06AB05 |
| Diphenhydramine | H1 antihistamine | R06AA02 |
| Chlorpheniramine | H1 antihistamine | R06AB04 |
| Brompheniramine | H1 antihistamine | R06AB01 |
| Cyproheptadine | H1 antihistamine | R06AX02 |
| Hydroxyzine | H1 antihistamine | N05BB01 |
| Promethazine | Antiemetic / antihistamine | R06AD02 |
| Dicyclomine | Antispasmodic | A03AA07 |
| Hyoscyamine | Antispasmodic | A03BA03 |
| Propantheline | Antispasmodic | A03AB05 |
| Glycopyrrolate (oral) | Antispasmodic | A03AB02 |
| Atropine (systemic) | Anticholinergic | A03BA01 |
| Scopolamine (hyoscine) | Anticholinergic | A04AD01 |
| Oxybutynin | Bladder antimuscarinic | G04BD04 |
| Tolterodine | Bladder antimuscarinic | G04BD07 |
| Solifenacin | Bladder antimuscarinic | G04BD08 |
| Darifenacin | Bladder antimuscarinic | G04BD10 |
| Trospium | Bladder antimuscarinic | G04BD09 |
| Fesoterodine | Bladder antimuscarinic | G04BD11 |
| Thioridazine | Antipsychotic | N05AC02 |
| Clozapine | Antipsychotic | N05AH02 |
| Olanzapine | Antipsychotic | N05AH03 |
| Benztropine | Antiparkinsonian anticholinergic | N04AC01 |
| Trihexyphenidyl | Antiparkinsonian anticholinergic | N04AA01 |

Abbreviations: ATC: anatomical therapeutic chemical

**Table S6**. Negative control outcomes used in the study

| **Category** | **ICD-10-CM codes** |
| --- | --- |
| Deviated nasal septum | J34.2 |
| Impacted cerumen | H61.2, H61.20, H61.21, H61.22, H61.23 |
| Regular astigmatism | H52.22, H52.221, H52.222, H52.223, H52.229 |

Abbreviations: ICD-10-CM: International Classification of Diseases System, 10th Revision, Clinical Modification; NCO: negative control outcome.

**Table S7**. Baseline characteristics of patients initiating treatment with GLP-1 RA versus DPP4i and SGLT2i before 1:1 PS matching using Penn Medicine EHR data*

| **Characteristics** | | **GLP-1 RA vs. DPP4i** | | |  | **GLP-1 RA vs. SGLT2i** | | |
| --- | --- | --- | --- | --- | --- | --- | --- | --- |
|  |  | **GLP-1 RA**  **(n =** **12178)** | **DPP4i**  **(n =** **11465)** | **SMD** |  | **GLP-1 RA**  **(n =** **11397)** | **SGLT2i**  **(n =** **18209)** | **SMD** |
| **Sex** | |  |  | 0.106 |  |  |  | 0.315 |
|  | Female | 6362 (52.2) | 5381 (46.9) |  |  | 6200 (54.4) | 7078 (38.9) |  |
|  | Male | 5816 (47.8) | 6084 (53.1) |  |  | 5197 (45.6) | 11131 (61.1) |  |
| **Mean age (SD), y** | | 63.33 (8.36) | 68.59 (10.09) | 0.568 |  | 63.39 (8.43) | 66.84 (9.32) | 0.388 |
| **Age** | |  |  | 0.428 |  |  |  | 0.312 |
|  | <65 years | 7050 (57.9) | 4241 (37.0) |  |  | 6594 (57.9) | 7726 (42.4) |  |
|  | ≥65 years | 5128 (42.1) | 7224 (63.0) |  |  | 4803 (42.1) | 10483 (57.6) |  |
| **Race/ethnicity** | |  |  | 0.277 |  |  |  | 0.190 |
|  | Asian | 369 (3.0) | 789 (6.9) |  |  | 327 (2.9) | 952 (5.2) |  |
|  | Hispanic | 501 (4.1) | 559 (4.9) |  |  | 480 (4.2) | 808 (4.4) |  |
|  | Non-Hispanic Black | 4224 (34.7) | 2792 (24.4) |  |  | 3996 (35.1) | 5118 (28.1) |  |
|  | Non-Hispanic White | 6377 (52.4) | 6438 (56.2) |  |  | 5945 (52.2) | 9942 (54.6) |  |
|  | Other/Unknown | 707 (5.8) | 887 (7.7) |  |  | 649 (5.7) | 1389 (7.6) |  |
| **Health insurance** | |  |  | 0.371 |  |  |  | 0.268 |
|  | Commercial | 5128 (42.1) | 3266 (28.5) |  |  | 4800 (42.1) | 5914 (32.5) |  |
|  | Medicaid | 1314 (10.8) | 735 (6.4) |  |  | 1241 (10.9) | 1410 (7.7) |  |
|  | Medicare | 4637 (38.1) | 6093 (53.1) |  |  | 4382 (38.4) | 8498 (46.7) |  |
|  | Self-Pay/Other | 1099 (9.0) | 1371 (12.0) |  |  | 974 (8.5) | 2387 (13.1) |  |
| **Site ID** | |  |  | 0.329 |  |  |  | 0.178 |
|  | 1 | 2635 (21.6) | 1908 (16.6) |  |  | 2434 (21.4) | 3468 (19.0) |  |
|  | 2 | 5839 (47.9) | 4852 (42.3) |  |  | 5374 (47.2) | 9053 (49.7) |  |
|  | 3 | 1577 (12.9) | 1131 (9.9) |  |  | 1521 (13.3) | 1653 (9.1) |  |
|  | 4 | 737 (6.1) | 1236 (10.8) |  |  | 738 (6.5) | 1539 (8.5) |  |
|  | 5 | 1152 (9.5) | 1933 (16.9) |  |  | 1108 (9.7) | 1931 (10.6) |  |
|  | Other | 238 (2.0) | 405 (3.5) |  |  | 222 (1.9) | 565 (3.1) |  |
| **Patient setting** | |  |  | 0.237 |  |  |  | 0.122 |
|  | Emergency | 51 (0.4) | 88 (0.8) |  |  | 50 (0.4) | 102 (0.6) |  |
|  | Inpatient | 390 (3.2) | 985 (8.6) |  |  | 346 (3.0) | 996 (5.5) |  |
|  | Others | 71 (0.6) | 80 (0.7) |  |  | 64 (0.6) | 96 (0.5) |  |
|  | Outpatient | 11666 (95.8) | 10312 (89.9) |  |  | 10937 (96.0) | 17015 (93.4) |  |
| **CCI** | |  |  | 0.369 |  |  |  | 0.095 |
|  | 0 | 1349 (11.1) | 2813 (24.5) |  |  | 1274 (11.2) | 2243 (12.3) |  |
|  | 1~2 | 1040 (8.5) | 596 (5.2) |  |  | 985 (8.6) | 1139 (6.3) |  |
|  | >=3 | 9789 (80.4) | 8056 (70.3) |  |  | 9138 (80.2) | 14827 (81.4) |  |
| **Emergency visits** | |  |  | 0.346 |  |  |  | 0.044 |
|  | 0 | 1282 (10.5) | 2670 (23.3) |  |  | 1206 (10.6) | 2125 (11.7) |  |
|  | 1~2 | 10089 (82.8) | 8114 (70.8) |  |  | 9412 (82.6) | 14974 (82.2) |  |
|  | >=3 | 807 (6.6) | 681 (5.9) |  |  | 779 (6.8) | 1110 (6.1) |  |
| **Inpatient visits** | |  |  | 0.358 |  |  |  | 0.157 |
|  | 0 | 1091 (9.0) | 2033 (17.7) |  |  | 1025 (9.0) | 1733 (9.5) |  |
|  | 1~2 | 9611 (78.9) | 7239 (63.1) |  |  | 9036 (79.3) | 13370 (73.4) |  |
|  | >=3 | 1476 (12.1) | 2193 (19.1) |  |  | 1336 (11.7) | 3106 (17.1) |  |
| **Outpatient visits** | |  |  | 0.171 |  |  |  | 0.109 |
|  | 0 | 317 (2.6) | 659 (5.7) |  |  | 281 (2.5) | 588 (3.2) |  |
|  | 1~2 | 859 (7.1) | 982 (8.6) |  |  | 762 (6.7) | 1693 (9.3) |  |
|  | >=3 | 11002 (90.3) | 9824 (85.7) |  |  | 10354 (90.8) | 15928 (87.5) |  |
| **Mean health examination results (SD)** | |  |  |  |  |  |  |  |
|  | BMI, *kg/m^2^* | 35.40 (8.02) | 30.99 (7.70) | 0.562 |  | 35.54 (8.07) | 32.12 (7.51) | 0.439 |
|  | Systolic blood pressure, *mmHg* | 132.38 (18.06) | 133.34 (19.81) | 0.051 |  | 132.72 (17.90) | 131.69 (19.53) | 0.055 |
|  | Diastolic blood pressure, *mmHg* | 77.54 (10.76) | 75.83 (11.26) | 0.156 |  | 77.71 (10.70) | 76.05 (11.48) | 0.150 |
|  | HbA1c, *%* | 8.36 (4.92) | 8.71 (4.07) | 0.078 |  | 8.34 (4.69) | 8.57 (3.51) | 0.053 |
|  | Non-fasting blood glucose, *mg/dL* | 163.55 (77.93) | 172.24 (73.70) | 0.115 |  | 166.54 (80.42) | 165.66 (73.81) | 0.011 |
|  | Alanine aminotransferase, *IU/L* | 26.84 (39.24) | 27.79 (59.14) | 0.019 |  | 26.48 (38.22) | 26.01 (41.53) | 0.012 |
|  | Aspartate aminotransferase, *IU/L* | 24.97 (46.53) | 26.36 (55.60) | 0.027 |  | 25.52 (51.78) | 26.42 (58.73) | 0.016 |
|  | Alkaline phosphatase, *IU/L* | 83.29 (37.94) | 84.26 (54.43) | 0.021 |  | 83.95 (44.75) | 85.65 (55.71) | 0.034 |
|  | eGFR, *mL/min/1.73 m^2^* | 76.39 (21.93) | 70.75 (24.79) | 0.241 |  | 76.63 (21.92) | 71.60 (23.49) | 0.221 |
|  | Creatinine, *mg/dL* | 1.03 (0.47) | 1.12 (0.63) | 0.156 |  | 1.03 (0.50) | 1.12 (0.52) | 0.179 |
|  | Total cholesterol, *mg/dL* | 162.07 (47.65) | 159.08 (48.24) | 0.062 |  | 162.36 (48.17) | 154.62 (51.47) | 0.155 |
|  | Triglyceride, *mg/dL* | 159.55 (103.17) | 156.08 (98.55) | 0.034 |  | 158.92 (100.19) | 156.71 (105.75) | 0.021 |
|  | HD cholesterol L, *mg/dL* | 46.96 (13.78) | 46.50 (14.52) | 0.032 |  | 47.30 (14.01) | 45.73 (14.00) | 0.112 |
|  | LDL cholesterol, *mg/dL* | 95.12 (73.06) | 91.41 (68.73) | 0.052 |  | 95.00 (70.51) | 89.37 (72.18) | 0.079 |
| **Diabetes-related conditions** | |  |  |  |  |  |  |  |
|  | Diabetic nephropathy | 1963 (16.1) | 2086 (18.2) | 0.055 |  | 1716 (15.1) | 4146 (22.8) | 0.198 |
|  | Diabetic retinopathy | 967 (7.9) | 595 (5.2) | 0.111 |  | 857 (7.5) | 1383 (7.6) | 0.003 |
|  | Diabetic neuropathy | 1777 (14.6) | 1379 (12.0) | 0.076 |  | 1647 (14.5) | 2686 (14.8) | 0.008 |
|  | Diabetic peripheral vascular disease | 756 (6.2) | 724 (6.3) | 0.004 |  | 656 (5.8) | 1597 (8.8) | 0.116 |
|  | Other specified complications | 5659 (46.5) | 4607 (40.2) | 0.127 |  | 5242 (46.0) | 8214 (45.1) | 0.018 |
|  | Other unspecified complications | 1418 (11.6) | 860 (7.5) | 0.141 |  | 1315 (11.5) | 1762 (9.7) | 0.060 |
| **Comorbid conditions** | |  |  |  |  |  |  |  |
|  | Hypertension | 10022 (82.3) | 9312 (81.2) | 0.028 |  | 9378 (82.3) | 15420 (84.7) | 0.065 |
|  | Heart failure | 1662 (13.6) | 1686 (14.7) | 0.030 |  | 1412 (12.4) | 4897 (26.9) | 0.371 |
|  | Cerebrovascular disease | 1361 (11.2) | 1654 (14.4) | 0.097 |  | 1242 (10.9) | 2835 (15.6) | 0.138 |
|  | Obesity | 6557 (53.8) | 3176 (27.7) | 0.552 |  | 6154 (54.0) | 6838 (37.6) | 0.335 |
|  | Dyslipidemia | 9672 (79.4) | 8803 (76.8) | 0.064 |  | 8989 (78.9) | 14985 (82.3) | 0.087 |
|  | Chronic kidney disease | 2138 (17.6) | 2568 (22.4) | 0.121 |  | 1894 (16.6) | 4862 (26.7) | 0.247 |
|  | Asthma | 1901 (15.6) | 1186 (10.3) | 0.157 |  | 1781 (15.6) | 2213 (12.2) | 0.101 |
|  | COPD | 1017 (8.4) | 1004 (8.8) | 0.015 |  | 935 (8.2) | 1985 (10.9) | 0.092 |
|  | Mild cognitive impairment | 114 (0.9) | 113 (1.0) | 0.005 |  | 110 (1.0) | 175 (1.0) | <0.001 |
|  | Parkinson’s disease | 55 (0.5) | 97 (0.8) | 0.049 |  | 55 (0.5) | 119 (0.7) | 0.023 |
|  | Anxiety disorder | 2828 (23.2) | 1935 (16.9) | 0.159 |  | 2678 (23.5) | 3541 (19.4) | 0.099 |
|  | Mood disorder | 2858 (23.5) | 1853 (16.2) | 0.184 |  | 2680 (23.5) | 3346 (18.4) | 0.127 |
|  | Substance use disorder | 1728 (14.2) | 1335 (11.6) | 0.076 |  | 1585 (13.9) | 2771 (15.2) | 0.037 |
|  | Schizophrenia | 84 (0.7) | 107 (0.9) | 0.027 |  | 76 (0.7) | 146 (0.8) | 0.016 |
|  | Sleep disorder | 4430 (36.4) | 2501 (21.8) | 0.325 |  | 4140 (36.3) | 5572 (30.6) | 0.122 |
|  | Vitamin b12 deficiency | 182 (1.5) | 140 (1.2) | 0.024 |  | 171 (1.5) | 303 (1.7) | 0.013 |
|  | Rheumatoid arthritis | 255 (2.1) | 238 (2.1) | 0.001 |  | 240 (2.1) | 385 (2.1) | 0.001 |
| **Concomitant drugs** | |  |  |  |  |  |  |  |
|  | Antidepressants | 1022 (8.4) | 98 (0.9) | 0.365 |  | 898 (7.9) | 202 (1.1) | 0.331 |
|  | Antipsychotics | 330 (2.7) | 28 (0.2) | 0.205 |  | 290 (2.5) | 63 (0.3) | 0.185 |
|  | ACEi/ARB | 1905 (15.6) | 203 (1.8) | 0.508 |  | 1637 (14.4) | 437 (2.4) | 0.442 |
|  | Beta blockers | 936 (7.7) | 95 (0.8) | 0.345 |  | 780 (6.8) | 208 (1.1) | 0.294 |
|  | Calcium-channel blockers | 1184 (9.7) | 103 (0.9) | 0.401 |  | 1015 (8.9) | 245 (1.3) | 0.348 |
|  | Corticosteroids | 758 (6.2) | 79 (0.7) | 0.307 |  | 665 (5.8) | 163 (0.9) | 0.277 |
|  | Diuretics | 1831 (15.0) | 177 (1.5) | 0.505 |  | 1568 (13.8) | 418 (2.3) | 0.432 |
|  | Lipid-lowering drugs | 2483 (20.4) | 264 (2.3) | 0.595 |  | 2130 (18.7) | 566 (3.1) | 0.516 |
|  | NSAIDs | 966 (7.9) | 68 (0.6) | 0.369 |  | 857 (7.5) | 181 (1.0) | 0.328 |
| **Other antidiabetic drugs** | |  |  |  |  |  |  |  |
|  | a-Glucosidase inhibitors | 19 (0.2) | 21 (0.2) | 0.007 |  | 23 (0.2) | 46 (0.3) | 0.011 |
|  | Insulin | 3642 (29.9) | 3408 (29.7) | 0.004 |  | 3380 (29.7) | 5542 (30.4) | 0.017 |
|  | Meglitinides | 199 (1.6) | 316 (2.8) | 0.077 |  | 206 (1.8) | 344 (1.9) | 0.006 |
|  | Metformin | 5778 (47.4) | 4631 (40.4) | 0.142 |  | 5502 (48.3) | 7902 (43.4) | 0.098 |
|  | Sulfonylureas | 1859 (15.3) | 2324 (20.3) | 0.131 |  | 1864 (16.4) | 3252 (17.9) | 0.040 |
|  | Thiazolidinediones | 270 (2.2) | 276 (2.4) | 0.013 |  | 264 (2.3) | 478 (2.6) | 0.020 |
|  | DPP4is | - | - | - |  | 944 (8.3) | 1571 (8.6) | 0.012 |
|  | SGLT2is | 1304 (10.7) | 905 (7.9) | 0.097 |  | - | - | - |

Abbreviations: ACEi: angiotensin-converting enzyme inhibitor; ARB: angiotensin-receptor blocker; BMI: body mass index; CCI: Charlson Comorbidity Index; COPD: chronic obstructive pulmonary disease; DPP4i: dipeptidyl peptidase-4 inhibitor; eGFR: estimated glomerular filtration rate; EHR: electronic health record; GLP-1 RA: glucagon-like peptide-1 receptor agonist; HDL: high-density lipoprotein; LDL: low-density lipoprotein; NSAID: nonsteroidal anti-inflammatory drug; PS: propensity score; SGLT2i: sodium-glucose cotransporter-2 inhibitor.

* Values are numbers (percentages) unless otherwise indicated.

**
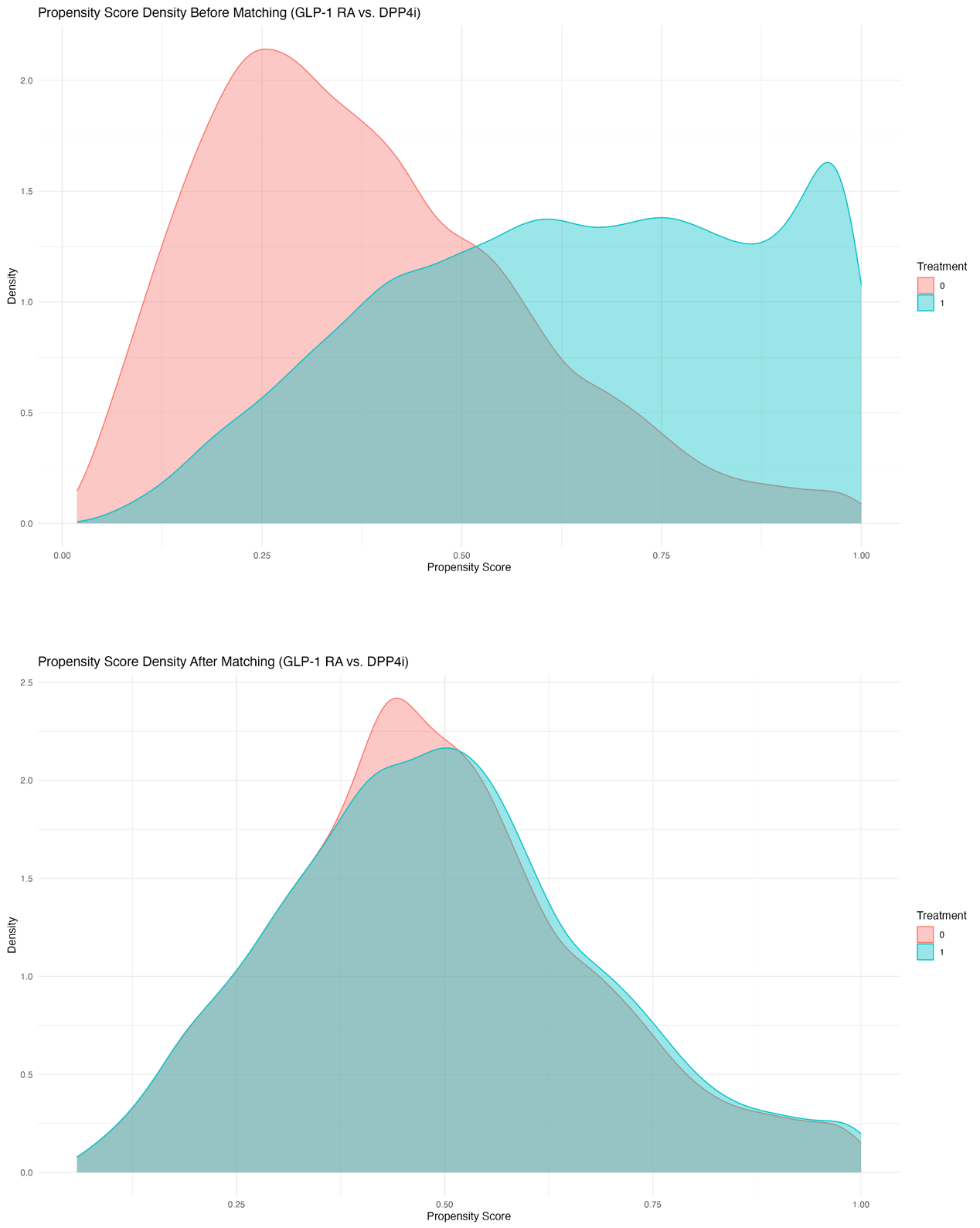
**

**Figure S1**. Propensity score density for GLP-1 RA (Treatment=1) versus DPP4i (Treatment=0) before and after 1:1 PS matching using Penn Medicine EHR data for primary analysis

**
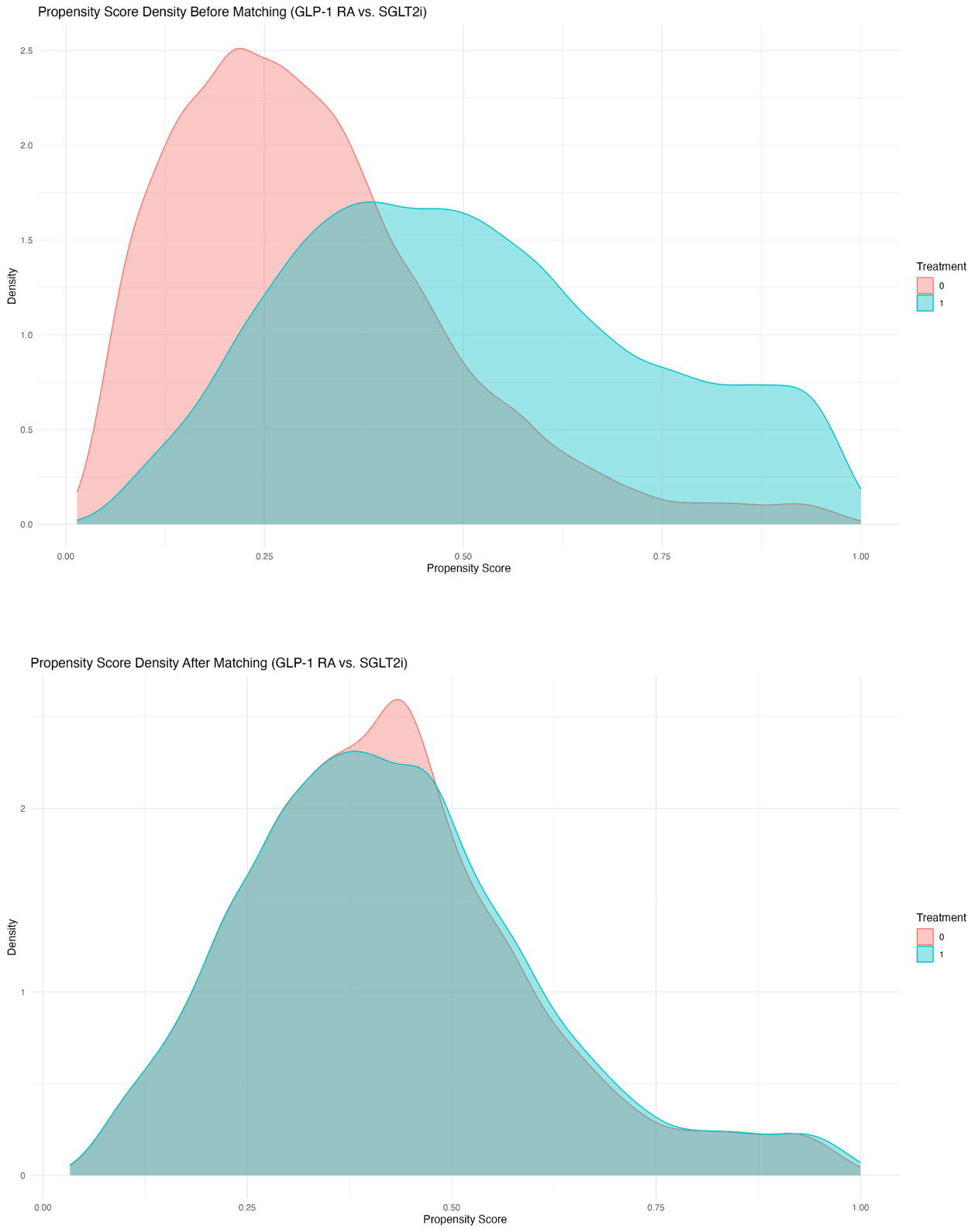
**

**Figure S2**. Propensity score density for GLP-1 RA (Treatment=1) versus SGLT2i (Treatment=0) before and after 1:1 PS matching using Penn Medicine EHR data for primary analysis

**Table S8**. Details of cohort construction for GLP-1 RA vs DPP4i cohorts in TriNetX

| **(1). GLP-1 RA cohort** | **Patients** | **HCOs** |
| --- | --- | --- |
| **Base Population** | 133,411,384 | 101 |
| **Group 1A: Include: patient using glucagon-like peptide-1 receptor agonists (GLP-1 RAs) -** The terms in this group occurred between Jan 1, 2019 and Sep 30, 2024. **Must Have:** GLP-1 RAs (at least 50 years old at event). **Group 1B -** Any instance of Group 1B occurred within 1 year on or before the first instance of Group 1A. **Must Have:** Type 2 diabetes mellitus and at least one visit. **Cannot Have:** End stage renal disease, Alzheimer's disease,  Vascular dementia, Dementia in other diseases classified elsewhere,  Unspecified dementia or Dipeptidyl peptidase 4 inhibitors (DPP4is) |  |  |
| **Final population** | **294,806** | **69** |
| **(2). DPP4i cohort** | **Patients** | **HCOs** |
| **Base Population** | 133,411,384 | 101 |
| **Group 1A: Include: patient using DPP4is -** The terms in this group occurred between Jan 1, 2019 and Sep 30, 2024. **Must Have:** DPP4is (at least 50 years old at event). **Group 1B -** Any instance of Group 1B occurred within 1 year on or before the first instance of Group 1A. **Must Have:** Type 2 diabetes mellitus and at least one visit. **Cannot Have:** End stage renal disease, Alzheimer's disease,  Vascular dementia, Dementia in other diseases classified elsewhere,  Unspecified dementia or GLP-1 RAs |  |  |
| **Final population** | **203,052** | **69** |

**Table S9**. Details of cohort construction for GLP-1 RA vs SGLT2i cohorts in TriNetX

| **(1). GLP-1 RA cohort** | **Patients** | **HCOs** |
| --- | --- | --- |
| **Base Population** | 133,411,384 | 101 |
| **Group 1A: Include: patient using glucagon-like peptide-1 receptor agonists (GLP-1 RAs) -** The terms in this group occurred between Jan 1, 2019 and Sep 30, 2024. **Must Have:** GLP-1 RAs (at least 50 years old at event). **Group 1B -** Any instance of Group 1B occurred within 1 year on or before the first instance of Group 1A. **Must Have:** Type 2 diabetes and at least one visit. **Cannot Have:** End stage renal disease, Alzheimer's disease,  Vascular dementia, Dementia in other diseases classified elsewhere,  Unspecified dementia or Sodium-glucose co-transporter 2 inhibitors (SGLT2is) |  |  |
| **Final population** | **262,349** | **69** |
| **(2). SGLT2i cohort** | **Patients** | **HCOs** |
| **Base Population** | 133,411,384 | 101 |
| **Group 1A: Include: patient using SGLT2is -** The terms in this group occurred between Jan 1, 2019 and Sep 30, 2024. **Must Have:** SGLT2is (at least 50 years old at event). **Group 1B -** Any instance of Group 1B occurred within 1 year on or before the first instance of Group 1A. **Must Have:** Type 2 diabetes and at least one visit. **Cannot Have:** End stage renal disease, Alzheimer's disease,  Vascular dementia, Dementia in other diseases classified elsewhere,  Unspecified dementia or GLP-1 RAs |  |  |
| **Final population** | **256,463** | **69** |

**Table S10**. Reasons for censoring in 1:1 PS-matched cohorts for GLP-1 RAs versus DPP4is and SGLT2is using Penn Medicine EHR data, N (%) ^*^

| **Censoring reason** | **GLP-1 RA vs. DPP4i**  **(n=6677 pairs)** | |  | **GLP-1 RA vs. SGLT2i**  **(n=8434 pairs)** | |
| --- | --- | --- | --- | --- | --- |
|  | **GLP-1 RA** | **DPP4i** |  | **GLP-1 RA** | **SGLT2i** |
| Death | 350 (5.24) | 302 (4.52) |  | 326 (3.87) | 263 (3.12) |
| End of study period | 2235 (33.47) | 2615 (39.16) |  | 3519 (41.72) | 4662 (55.28) |
| Incident dementia | 115 (1.72) | 90 (1.35) |  | 106 (1.26) | 56 (0.66) |
| Last recorded healthcare encounter | 3977 (59.56) | 3670 (54.96) |  | 4483 (53.15) | 3453 (40.94) |

Abbreviations: DPP4i: dipeptidyl peptidase-4 inhibitor; GLP-1 RA: glucagon-like peptide-1 receptor agonist; HR: hazard ratio; IR: incidence rate; PS: propensity score; SGLT2i: sodium-glucose cotransporter-2 inhibitor.

* Percentages are calculated within treatment groups. “Last recorded healthcare encounter” reflects disengagement from the health system prior to dementia, death, or study end.


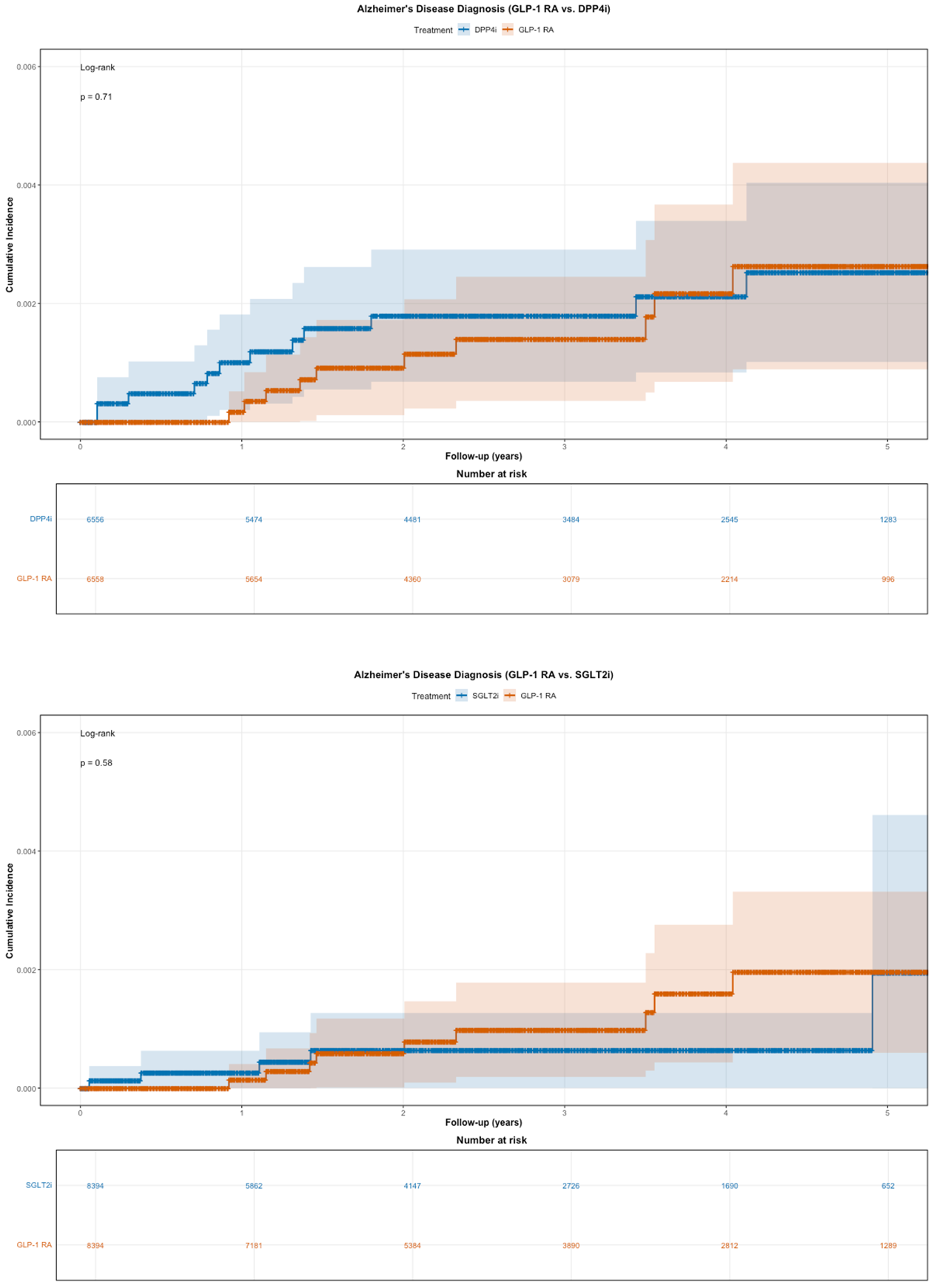


**Figure S3**. Cumulative incidence of Alzheimer’s disease over time in the 1:1 PS-matched cohorts for GLP-1 RAs vs. DPP4is (top) and GLP-1 RAs vs. SGLT2is (bottom) using Penn Medicine EHR data. The figure includes a risk table beneath a Kaplan-Meier curve, showing each cohort's number of patients at risk every year.

Abbreviations: DPP4i:dipeptidyl peptidase-4 inhibitor; GLP-1 RA: glucagon-like peptide-1 receptor agonist; PS: propensity score; SGLT2i: sodium-glucose cotransporter-2 inhibitor.


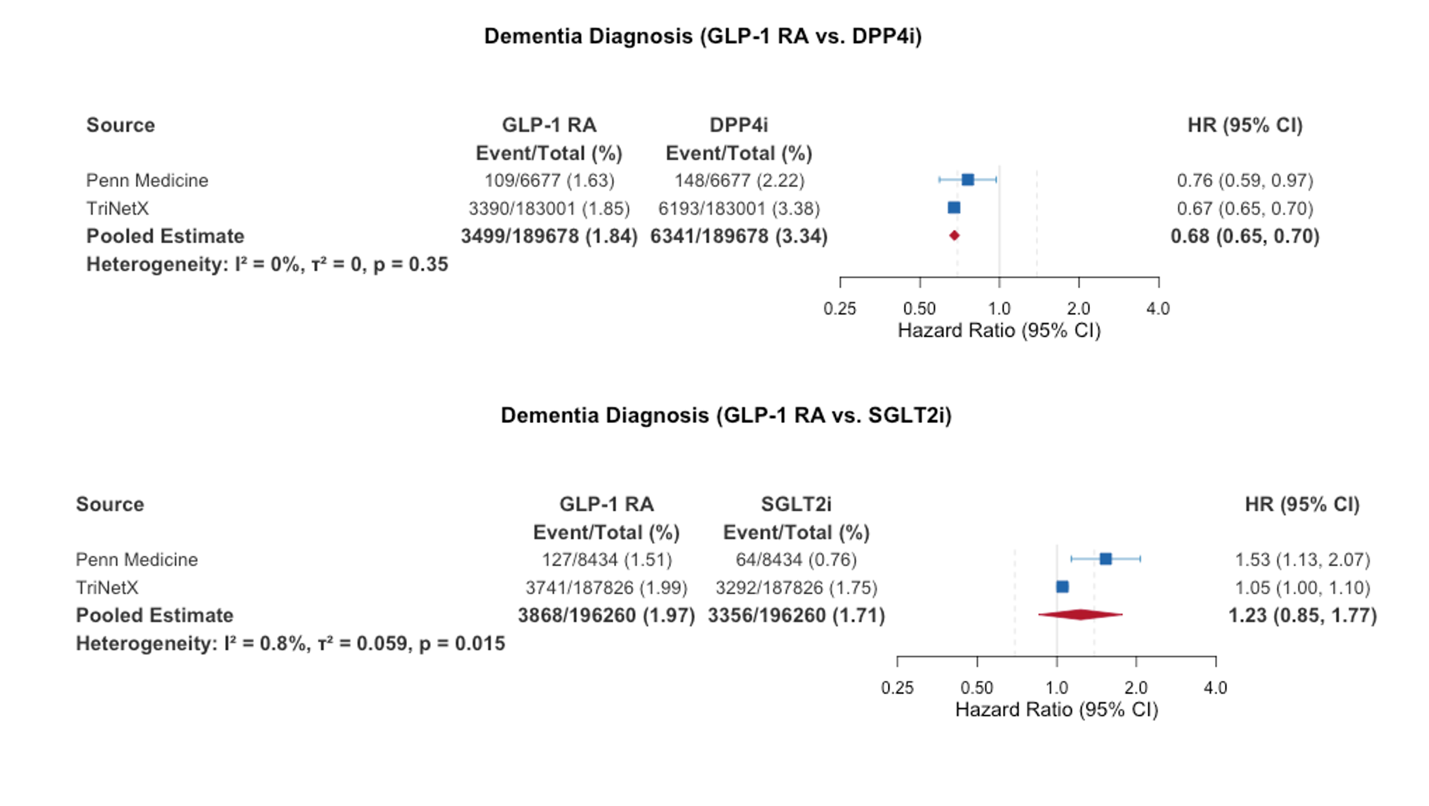


**Figure S4**. Pooled estimate of hazard ratios for dementia diagnosis in the 1:1 PS-matched cohorts for GLP-1 RAs vs. DPP4is (top) and GLP-1 RAs vs. SGLT2is (bottom) using data from Penn Medicine and TriNetX.

Abbreviations: DPP4i:dipeptidyl peptidase-4 inhibitor; GLP-1 RA: glucagon-like peptide-1 receptor agonist; HR: hazard ratio; SGLT2i: sodium-glucose cotransporter-2 inhibitor.


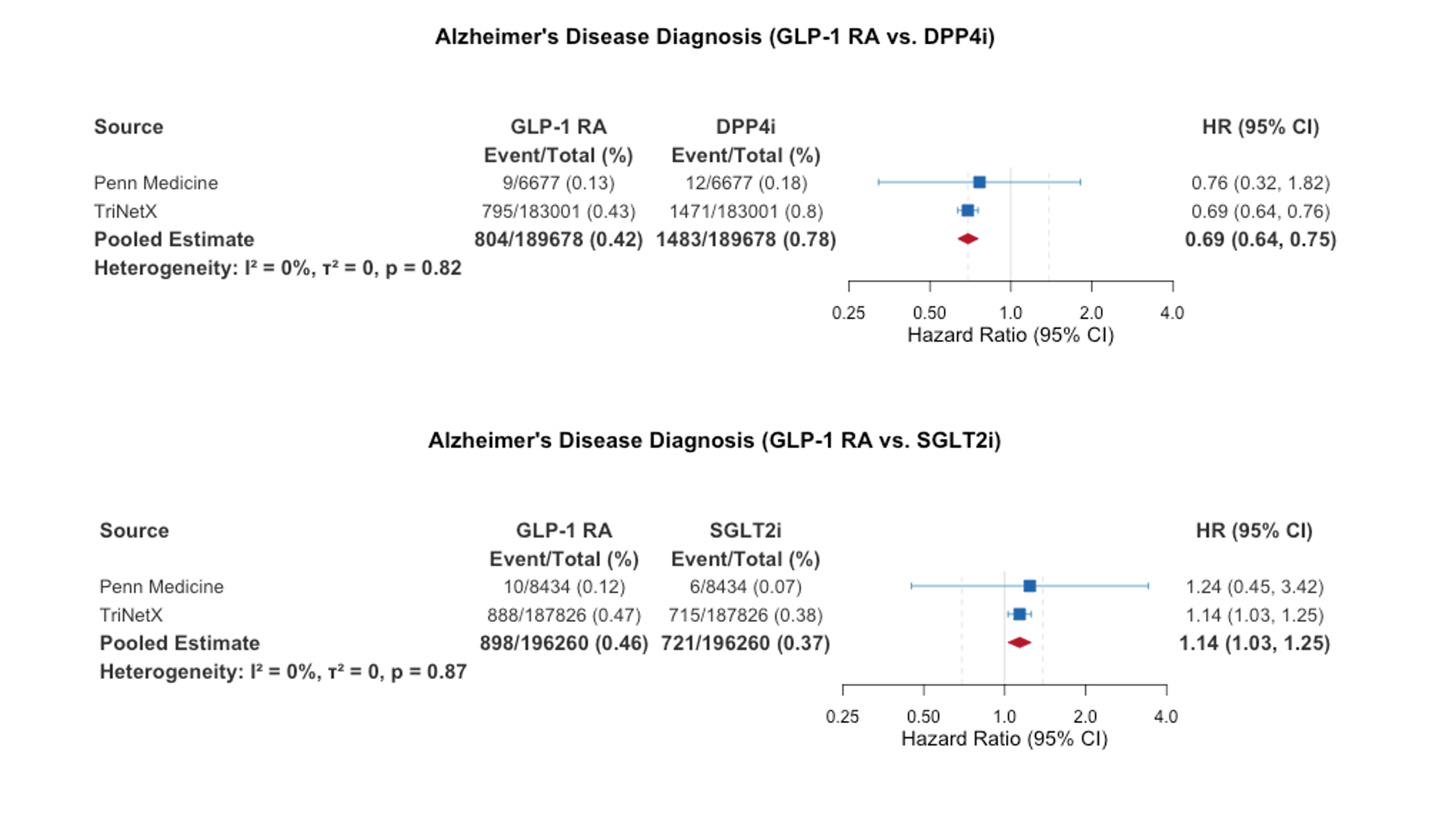


**Figure S5**. Pooled estimate of hazard ratios of Alzheimer’s disease diagnosis in the 1:1 PS-matched cohorts for GLP-1 RAs vs. DPP4is (top) and GLP-1 RAs vs. SGLT2is (bottom) using data from Penn Medicine and TriNetX.

Abbreviations: DPP4i:dipeptidyl peptidase-4 inhibitor; GLP-1 RA: glucagon-like peptide-1 receptor agonist; HR: hazard ratio; SGLT2i: sodium-glucose cotransporter-2 inhibitor.

**Table S11**. Sensitivity analysis using a stricter dementia outcome in 1:1 PS-matched cohorts for GLP-1 RAs versus DPP4is and SGLT2is using Penn Medicine EHR data^*^

| **Outcomes** | **GLP-1 RA vs. DPP4i** | |  | **GLP-1 RA vs. SGLT2i** | |
| --- | --- | --- | --- | --- | --- |
|  | **GLP-1 RA** | **DPP4i** |  | **GLP-1 RA** | **SGLT2i** |
| Events/Patients at risk, n/N | 12/6677 | 32/6677 |  | 11/8434 | 8/8434 |
| Median follow-up (IQR), y | 2.95 (1.70, 4.68) | 3.38 (1.63, 4.68) |  | 2.87 (1.53, 4.66) | 2.01 (0.78 3.65) |
| IR per 1000 person-years | 0.59 | 0.59 |  | 0.44 | 0.41 |
| HR (95% CI) | 0.38 (0.19, 0.73) | Reference |  | 1.18 (0.47, 2.93) | Reference |

Abbreviations: DPP4i: dipeptidyl peptidase-4 inhibitor; GLP-1 RA: glucagon-like peptide-1 receptor agonist; HR: hazard ratio; IR: incidence rate; PS: propensity score; SGLT2i: sodium-glucose cotransporter-2 inhibitor.

* A stricter dementia outcome was defined as at least one dementia diagnosis after the index date and a dementia-specific medication within 12 months, with the event date defined as the date of the first post-index dementia diagnosis.

**Table S12**. Sensitivity analysis adjusting baseline strong anticholinergic medications in 1:1 PS-matched cohorts for GLP-1 RAs versus DPP4is and SGLT2is using Penn Medicine EHR data^*^

| **Outcomes** | **GLP-1 RA vs. DPP4i** | |  | **GLP-1 RA vs. SGLT2i** | |
| --- | --- | --- | --- | --- | --- |
|  | **GLP-1 RA** | **DPP4i** |  | **GLP-1 RA** | **SGLT2i** |
| Events/Patients at risk, n/N | 100/6560 | 137/6560 |  | 128/8427 | 67/8427 |
| Median follow-up (IQR), y | 2.95 (1.60, 4.67) | 2.95 (1.54, 4.67) |  | 2.70 (1.47, 4.60) | 1.93 (0.73, 3.54) |
| IR per 1000 person-years | 5.04 | 6.93 |  | 5.21 | 3.55 |
| HR (95% CI) | 0.73 (0.56, 0.94) | Reference |  | 1.46 (1.08, 1.96) | Reference |

Abbreviations: DPP4i: dipeptidyl peptidase-4 inhibitor; GLP-1 RA: glucagon-like peptide-1 receptor agonist; HR: hazard ratio; IR: incidence rate; PS: propensity score; SGLT2i: sodium-glucose cotransporter-2 inhibitor.

* We included anticholinergic medications into our covariate set using the Anticholinergic Cognitive Burden (ACB), focusing on ACB 3-point (strong anticholinergics) agents commonly examined in dementia research.

**Table S13**. Sensitivity analysis using a fixed 2-year follow-up window in 1:1 PS-matched cohorts for GLP-1 RAs versus DPP4is and SGLT2is using Penn Medicine EHR data^*^

| **Outcomes** | **GLP-1 RA vs. DPP4i** | |  | **GLP-1 RA vs. SGLT2i** | |
| --- | --- | --- | --- | --- | --- |
|  | **GLP-1 RA** | **DPP4i** |  | **GLP-1 RA** | **SGLT2i** |
| Events/Patients at risk, n/N | 60/6677 | 85/6677 |  | 68/8434 | 44/8434 |
| Median follow-up (IQR), y | 2 (2, 2) | 2 (2, 2) |  | 2 (2, 2) | 2 (2, 2) |
| IR per 1000 person-years | 4.58 | 6.52 |  | 4.09 | 2.65 |
| HR (95% CI) | 0.70 (0.50, 0.98) | Reference |  | 1.55 (1.06, 2.26) | Reference |

Abbreviations: DPP4i: dipeptidyl peptidase-4 inhibitor; GLP-1 RA: glucagon-like peptide-1 receptor agonist; HR: hazard ratio; IR: incidence rate; PS: propensity score; SGLT2i: sodium-glucose cotransporter-2 inhibitor.

* We conducted a fixed follow-up sensitivity analysis in which each participant was censored at 2 years after cohort entry to ensure equal follow-up time across treatment groups.

**Table S14**. Sensitivity analysis for other dementia subtypes in 1:1 PS-matched cohorts for GLP-1 RAs versus DPP4is and SGLT2is using Penn Medicine EHR data^*^

| **Other dementia subtypes** | | **GLP-1 RA vs. DPP4i** | |  | **GLP-1 RA vs. SGLT2i** | |
| --- | --- | --- | --- | --- | --- | --- |
|  |  | **GLP-1 RA** | **DPP4i** |  | **GLP-1 RA** | **SGLT2i** |
| Vascular dementia | |  |  |  |  |  |
|  | Events/Patients at risk, n/N | 22/6677 | 15/6677 |  | 26/8434 | 12/8434 |
|  | Median follow-up (IQR), y | 2.34 (1.24, 4.04) | 2.50 (1.11, 4.32) |  | 2.35 (1.22, 4.14) | 1.59 (0.54, 3.12) |
|  | IR per 1000 person-years | 1.28 | 0.84 |  | 1.19 | 0.73 |
|  | HR (95% CI) | 1.56 (0.81, 3.00) | Reference |  | 1.64 (0.82, 3.245 | Reference |
|  |  |  |  |  |  |  |
| Mixed/other dementia | |  |  |  |  |  |
|  | Events/Patients at risk, n/N | 78/6677 | 121/6677 |  | 91/8434 | 46/8434 |
|  | Median follow-up (IQR), y | 2.34 (1.24, 4.02) | 2.48 (1.09, 4.30) |  | 2.34 (1.22, 4.14) | 1.59 (0.53, 3.12) |
|  | IR per 1000 person-years | 4.57 | 6.84 |  | 4.18 | 2.79 |
|  | HR (95% CI) | 0.679 (0.511, 0.903) | Reference |  | 1.46 (1.02, 2.08) | Reference |

Abbreviations: DPP4i: dipeptidyl peptidase-4 inhibitor; GLP-1 RA: glucagon-like peptide-1 receptor agonist; HR: hazard ratio; IR: incidence rate; PS: propensity score; SGLT2i: sodium-glucose cotransporter-2 inhibitor.

* We conducted sensitivity analysis by using vascular dementia and mixed/other dementia as outcomes to explore more dementia subtypes.

**Table S15**. Negative control outcomes analysis in 1:1 PS-matched cohorts for GLP-1 RAs versus DPP4is and SGLT2is using Penn Medicine EHR data^*^

| **Other dementia subtypes** | | **GLP-1 RA vs. DPP4i** | |  | **GLP-1 RA vs. SGLT2i** | |
| --- | --- | --- | --- | --- | --- | --- |
|  |  | **GLP-1 RA** | **DPP4i** |  | **GLP-1 RA** | **SGLT2i** |
| Deviated nasal septum | |  |  |  |  |  |
|  | Events/Patients at risk, n/N | 111/6610 | 106/6610 |  | 138/8332 | 102/8332 |
|  | Median follow-up (IQR), y | 2.30 (1.22, 4.01) | 2.47 (1.10, 4.28) |  | 2.33 (1.21, 4.13) | 1.57 (0.53, 3.08) |
|  | IR per 1000 person-years | 6.61 | 6.04 |  | 6.43 | 6.323 |
|  | HR (95% CI) | 1.13 (0.86, 1.47) | Reference |  | 0.97 (0.75, 1.26) | Reference |
|  |  |  |  |  |  |  |
| Impacted cerumen | |  |  |  |  |  |
|  | Events/Patients at risk, n/N | 224/6462 | 264/6462 |  | 309/8120 | 220/8120 |
|  | Median follow-up (IQR), y | 2.28 (1.167, 4.00) | 2.4 (1.01, 4.22) |  | 2.27 (1.18, 4.06) | 1.54 (0.53, 3.07) |
|  | IR per 1000 person-years | 13.83 | 15.72 |  | 15.00 | 14.07 |
|  | HR (95% CI) | 0.89 (0.75, 1.07) | Reference |  | 1.03 (0.87, 1.22) | Reference |
|  |  |  |  |  |  |  |
| Regular astigmatism | |  |  |  |  |  |
|  | Events/Patients at risk, n/N | 67/6572 | 71/6572 |  | 106/8265 | 60/8265 |
|  | Median follow-up (IQR), y | 2.32 (1.22, 4.06) | 2.45 (1.06, 4.21) |  | 2.32 (1.20, 4.14) | 1.57 (0.53, 3.11) |
|  | IR per 1000 person-years | 3.99 | 4.11 |  | 4.978 | 3.73 |
|  | HR (95% CI) | 0.97 (0.69, 1.35) | Reference |  | 1.33 (0.97, 1.83) | Reference |

Abbreviations: DPP4i: dipeptidyl peptidase-4 inhibitor; GLP-1 RA: glucagon-like peptide-1 receptor agonist; HR: hazard ratio; IR: incidence rate; NCO: negative control outcomes; PS: propensity score; SGLT2i: sodium-glucose cotransporter-2 inhibitor.


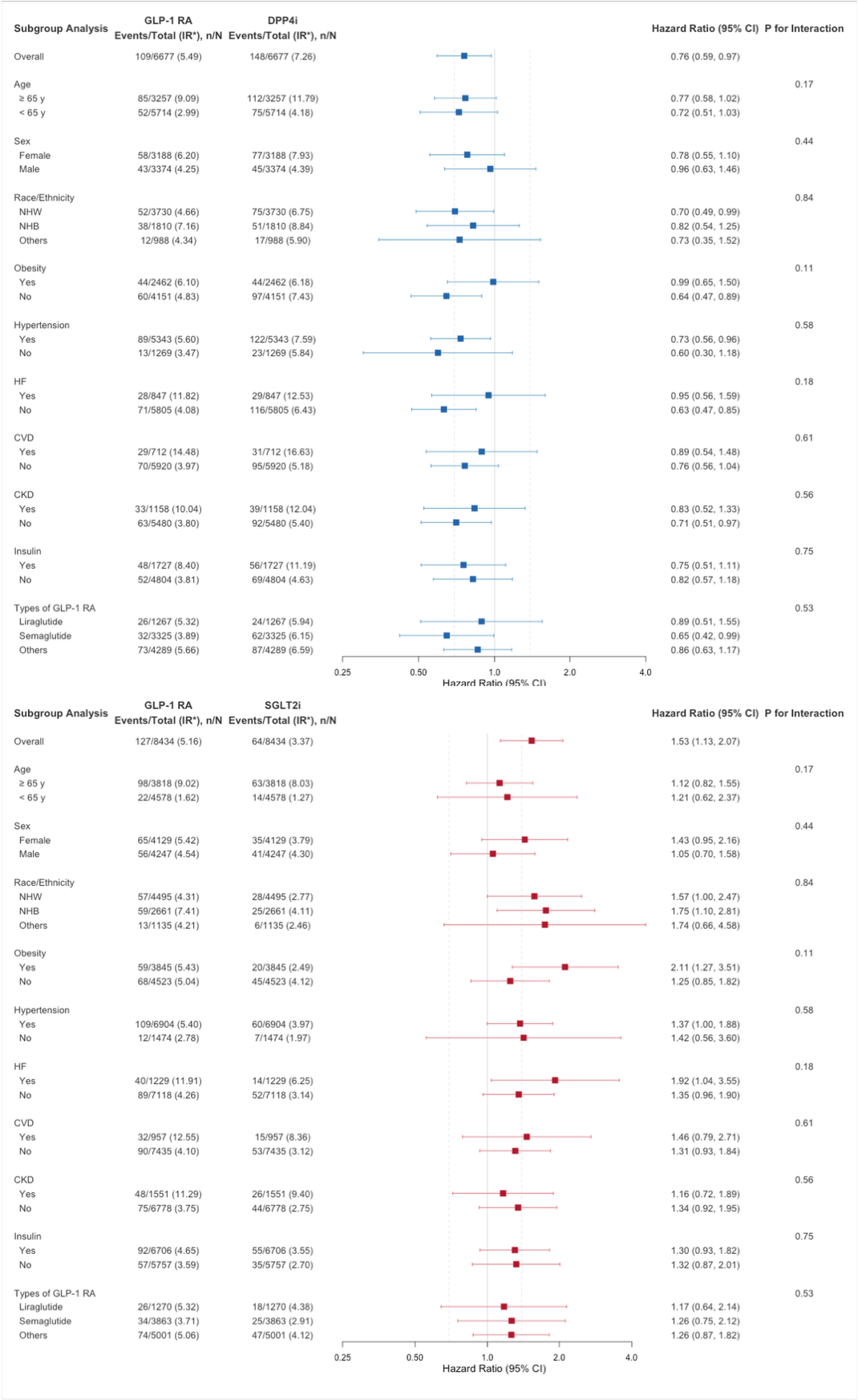


**Figure S6**. Subgroup analyses for dementia diagnosis in the 1:1 PS-matched cohorts for GLP-1 RAs vs. DPP4is (top) and GLP-1 RAs vs. SGLT2is (bottom) using Penn Medicine EHR data

Abbreviations: CKD: chronic kidney disease; CVD: cerebrovascular disease; DPP4i: dipeptidyl peptidase-4 inhibitor; GLP-1 RA: glucagon-like peptide-1 receptor agonist; HF: heart failure; HR: hazard ratio; IR: incidence rate; NHB: non-Hispanic Black; NHW: non-Hispanic White; PS: propensity score; SGLT2i: sodium-glucose cotransporter-2 inhibitor.

* Per 1000 person-years.
